# The *Drosophila* histone methyltransferase SET1 coordinates multiple signaling pathways in regulating male germline stem cell maintenance and differentiation

**DOI:** 10.1242/dev.202729

**Published:** 2024-08-09

**Authors:** Velinda Vidaurre, Annabelle Song, Taibo Li, Wai Lim Ku, Keji Zhao, Jiang Qian, Xin Chen

**Affiliations:** ^1^ Department of Biology, The Johns Hopkins University, Baltimore, MD 21218, USA; ^2^ Laboratory of Epigenome Biology, Systems Biology Center, National Heart, Lung and Blood Institute, NIH, Bethesda, MD 20814, USA; ^3^ Department of Ophthalmology, The Johns Hopkins University School of Medicine, Baltimore, MD 21287, USA; ^4^ Howard Hughes Medical Institute, 4000 Jones Bridge Road, Chevy Chase, MD 20815, USA

**Keywords:** Germline stem cell, Maintenance, Differentiation, Chromatin, Histone modification, Epigenetic, Signaling pathway, Gene expression

## Abstract

Many tissue-specific adult stem cell lineages maintain a balance between proliferation and differentiation. Here, we study how the H3K4me3 methyltransferase Set1 regulates early-stage male germ cells in *Drosophila*. Early-stage germline-specific knockdown of *Set1* results in temporally progressive defects, arising as germ cell loss and developing into overpopulated early-stage germ cells. These germline defects also impact the niche architecture and cyst stem cell lineage non-cell-autonomously. Additionally, wild-type Set1, but not the catalytically inactive Set1, rescues the *Set1* knockdown phenotypes, highlighting the functional importance of the methyltransferase activity of Set1. Further, RNA-sequencing experiments reveal key signaling pathway components, such as the JAK-STAT pathway gene *Stat92E* and the BMP pathway gene *Mad*, which are upregulated upon *Set1* knockdown. Genetic interaction assays support the functional relationships between *Set1* and JAK-STAT or BMP pathways, as both *Stat92E* and *Mad* mutations suppress the *Set1* knockdown phenotypes. These findings enhance our understanding of the balance between proliferation and differentiation in an adult stem cell lineage. The phenotype of germ cell loss followed by over-proliferation when inhibiting a histone methyltransferase also raises concerns about using their inhibitors in cancer therapy.

## Teaser

**Summary:** Set1 maintains germline activity by ensuring proper JAK-STAT and BMP signaling in the *Drosophila* testis.

## INTRODUCTION

In multicellular organisms, homeostasis and regeneration of many tissues largely depend on adult stem cells. These endogenous stem cells often undergo asymmetric cell divisions to allow both the stem cell to maintain its own population through self-renewal and the differentiation process to replace cells lost under physiological and pathological conditions (Kahney et al., 2019; Knoblich, 2008; Morrison and Spradling, 2008; Venkei and Yamashita, 2018). Disruption of these processes can potentially result in the misregulation of stem cell activity, leading to cancer or tissue degeneration (Clevers, 2005; Knoblich, 2010; Morrison and Kimble, 2006; Zion et al., 2020). There are two main modes of dysregulation in adult stem cell lineages that can result in the unrestrained cell proliferation that underlies tumorigenesis: constraints of normal stem cell expansion may be compromised or inactivated. This situation could occur if the dependence of stem cells on the niche is disrupted, resulting in a niche-independent overpopulation of stem cells or enhancement of stem cell activities. Alternatively, the transit-amplifying cells could fail to exit proliferation and enter the terminal differentiation program (Clarke and Fuller, 2006; Mukherjee et al., 2015; Sell, 2010; Zhang and Hsu, 2017).

**Fig. 1.**
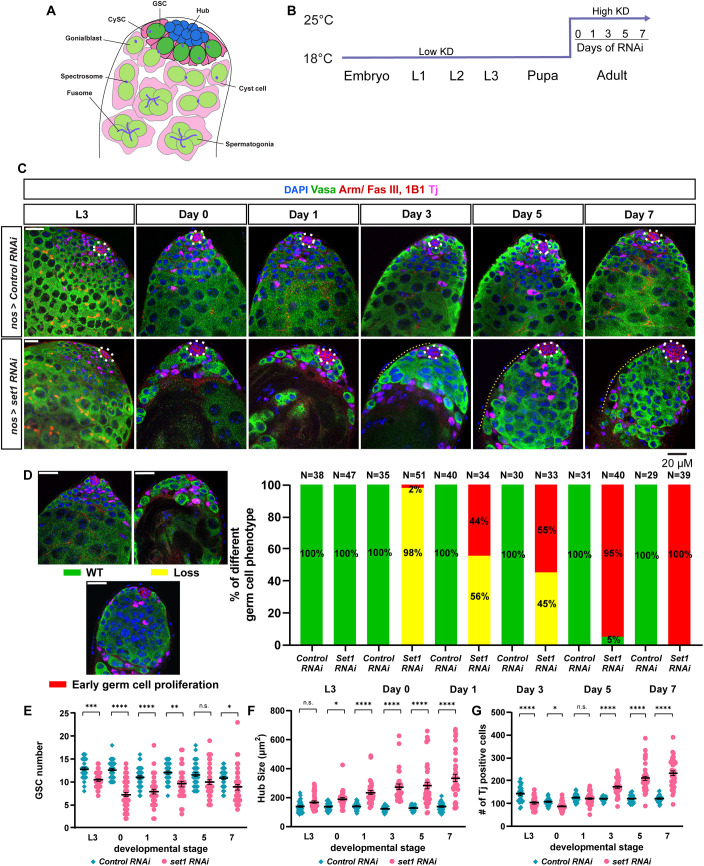
***Drosophila Set1* is required cell-autonomously for germline maintenance and differentiation.** (A) Schematic of the tip of the *Drosophila* testis. CySC, cyst stem cell; GSC, germline stem cell. (B) *nos>Control RNAi* (Ctrl KD) and *nos>set1 RNAi* (*Set1* KD) flies were grown at 18°C until eclosion, and then shifted to 25°C for 0, 1, 3, 5 or 7 days. (C) Representative Ctrl KD and *Set1* KD testes at the third instar larval stage (L3), day 0, 1, 3, 5 and 7 post-eclosion stained with the GSC lineage marker Vasa (green), Arm (red) or Fas III (red) for the hub area (white dashed outline), 1B1 for the spectrosome and fusome, and Tj (magenta) for the CySC lineage cells. Yellow dotted lines indicate overpopulated early germ cells. (D) Quantification of the percentage of testes with the representative germline phenotypes of germ cell loss (yellow) and early-germline overpopulation (red), present in Ctrl KD and *Set1* KD testes at L3, as well as 0, 1, 3, 5 and 7 days post-eclosion. (E) Quantification of GSC number for Ctrl KD testes (L3: *n*=38; day 0: *n*=35; day 1: *n*=40; day 3: *n*=30; day 5: *n*=31; day 7: *n*=29) and *Set1* KD testes (L3: *n*=47; day 0: *n*=51; day 1: *n*=34; day 3: *n*=33; day 5: *n*=40; day 7: *n*=39). See also Table S1. (F) Quantification of hub size for Ctrl KD and *Set1* KD testes. See also Table S2. (G) Quantification of early cyst cell number for Ctrl KD and *Set1* KD testes. See also Table S3. For E-G, individual data points and mean values are shown. Error bars represent s.e.m. ****P<10^−4^, ***P<10^−3^, **P<0.01, *P<0.05; n.s., not significant. Two-way ANOVA with interaction and Šidák multiple comparison test was used to compare two individual datasets with each other that have two independent variables. Scale bars: 20 μm.

*Drosophila* spermatogenesis is an excellent model system in which to study stem cell proliferation and differentiation (Davies and Fuller, 2008; Fuller, 1993; Gleason et al., 2018). Spermatogenesis is initiated with asymmetric division of the germline stem cell (GSC) to produce a self-renewed GSC and a gonialblast (Fig. 1A). The gonialblast undergoes four rounds of transit-amplifying mitotic divisions as spermatogonial cells. After mitosis, the 16 spermatogonial cells enter meiosis with a prolonged G2 phase as the primary spermatocytes. In addition to the GSC-derived germline lineage, the testis also has at least two somatic cell populations: the hub cells and the cyst cells (de Cuevas and Matunis, 2011). The hub cells are post-mitotic and they support the GSCs and the cyst stem cells (CySCs) (Kiger et al., 2001; Leatherman, 2013; Leatherman and Dinardo, 2010; Losick et al., 2011; Tran et al., 2000; Yamashita et al., 2003). The CySCs give rise to the cyst cells that support the germ cells throughout spermatogenesis (Leatherman and Dinardo, 2008; Lim and Fuller, 2012). The CySC also undergoes an asymmetric cell division to produce a self-renewed CySC and a cyst cell, which never divides again (Cheng et al., 2011). Two cyst cells encapsulate the germ cells as they divide and differentiate (Fig. 1A) (Fuller and Spradling, 2007; Matunis et al., 2012; Spradling et al., 2011).

Two important signaling pathways involved in the maintenance of the GSC niche are the JAnus Kinase Signal Transducer and Activator of Transcription (JAK-STAT) and Bone Morphogenetic Protein (BMP) signaling pathways (Amoyel et al., 2014; Herrera and Bach, 2019; Inaba et al., 2015; Kiger et al., 2001; Leatherman and Dinardo, 2008, 2010; Shivdasani and Ingham, 2003; Tulina and Matunis, 2001). Furthermore, epigenetic mechanisms, such as chromatin remodeling and histone modifications, have been shown to play important roles in the male germline lineage (Cherry and Matunis, 2010; Eun et al., 2017; Feng et al., 2018; Tarayrah and Chen, 2013; Tarayrah et al., 2015). In particular, multiple examples have shown the interplay between extrinsic signaling pathways and intrinsic epigenetic mechanisms in the *Drosophila* adult testes (Feng et al., 2017; Gleason and Chen, 2023; Vidaurre and Chen, 2021). For example, previous studies have shown that the H3K27me3 histone demethylase, Ubiquitously transcribed Tetratricopeptiderepeat gene on the X chromosome (UTX), acts as a negative regulator of JAK-STAT signaling by maintaining the transcription of *Socs36E*, proper expression and function of which maintain the balance between GSCs and CySCs (Amoyel et al., 2016; Issigonis et al., 2009; Singh et al., 2010; Tarayrah et al., 2013). Moreover, genes of the Epidermal Growth Factor (EGF) signaling pathway may be directly regulated by the H3K27me3 methyltransferase Enhancer of zestes [E(z)] in cyst cells, to ensure proper germ cell identity (Eun et al., 2014). Additionally, the *Drosophila* demethylase for H3K4me3, Little imaginal disc (Lid; Lysine demethylase 5, Kdm5), regulates JAK-STAT signaling in the male germline to maintain GSC activity and proliferation (Tarayrah et al., 2015). However, unlike the methyltransferase and demethylase for H3K27me3, the methyltransferases for H3K4me3 have not been studied in the *Drosophila* male germline.

SET domain containing 1 (Set1) is the main H3K4me3 methyltransferase in *Drosophila*, and its function is conserved from yeast to mammals (Lee et al., 2007a; Simonet et al., 2007). The post-translational modification of H3K4me3 has previously been shown to correlate with active transcription and is enriched at the promoter and the 5′ coding regions of genes (Bernstein et al., 2002; Heintzman et al., 2007; Pokholok et al., 2005; Santos-Rosa et al., 2002). The role of H3K4me3 in activating transcription is not fully understood, although it is thought that H3K4me3 acts as a docking scaffold for the transcription pre-initiation complex and chromatin remodeling complexes (Vermeulen et al., 2007; Wysocka et al., 2006). In yeast, Set1 catalyzes all methylation forms of H3K4 (i.e. H3K4me1/2/3) and its catalytic activity is modulated via a multi-subunit protein complex known as the Complex of proteins associated with Set1 (COMPASS) (Dehe et al., 2005; Miller et al., 2001; Nagy et al., 2002; Roguev et al., 2001). The *Drosophila* Set1 also interacts with the COMPASS complex to catalyze H3K4 di- and tri-methylation (H3K4me2/3) at the promoter proximal regions (Ardehali et al., 2011; Hallson et al., 2012; Mohan et al., 2011). In the *Drosophila* female germline, it has been shown that Set1 regulates GSC maintenance and differentiation; however, the mechanism has not been fully determined (Xuan et al., 2013; Yan et al., 2014).

Here, we use a series of molecular genetics and cell biology tools to study the roles of Set1 in the *Drosophila* testis. We found that Set1 is required for the normal function of early-stage male germ cells by regulating several key signaling pathways in a methyl-transferase-dependent manner. This study provides an example of how the intrinsic epigenetic mechanisms cooperate with the extrinsic signaling pathways to determine and maintain stem cell fate.

## RESULTS

### *Drosophila* Set1 regulates germline survival and proper germ cell differentiation

To explore the function of Set1 in the *Drosophila* male germline, a short hairpin RNA (shRNA or RNAi) specifically targeting the coding sequence of the *Set1* gene driven by the early-stage germline driver *nanos-Gal4* (*nos-Gal4*) (Van Doren et al., 1998) was employed for cell type-specific knockdown (KD) experiments. The *Set1* null allele is lethal at the pupal stage and conventional mosaic analysis cannot be performed owing to the genomic location of the *Set1* gene, which is very close to the centromere of chromosome 3. Therefore, the KD strategy is required to interrogate its roles *in vivo* (Ardehali et al., 2011; Hallson et al., 2012). In *nos>set1 RNAi* (*Set1* KD) testes, H3K4me3 signal was greatly reduced in the early-stage germ cells, from GSCs to spermatogonial cells (Fig. S1B), compared with germ cells at the comparable stages in the *nos>mCherry RNAi* testes (*nos>Control RNAi* or Ctrl KD; Fig. S1A)*.* In addition, in both *Set1* KD and Ctrl KD testes, H3K4me3 signals were present in the somatic gonadal cells, indicating germline-specific and efficient inactivation of the methyltransferase function of Set1 (Fig. S1).

To investigate potential defects in the testis, a time course experiment was performed whereby *Set1* KD and Ctrl KD flies were grown at 18°C until eclosion and then shifted to 25°C for 0, 1, 3, 5 or 7 days (Fig. 1B). We also examined an earlier development stage at the third instar larvae (L3), when male GSCs and their niche have already been established (Le Bras and Van Doren, 2006). At L3, 100% of *Set1* KD and Ctrl KD testes displayed normal morphology (Fig. 1C,D). At day 0 and day 1 post-eclosion, 98% and 56%, respectively, of the *Set1* KD testes, showed a germ cell loss phenotype compared with none of the Ctrl KD testes (Fig. 1C,D). At day 3 post-eclosion, although 45% of *Set1* KD testes had germline loss, 55% had an early germ cell overpopulation phenotype, whereby the germ cells surrounding the hub formed a large, disorganized cluster with very few intercalating cyst cells and were often devoid of or had very few late-stage spermatocytes (Fig. 1C,D). By days 5 and 7, 95% and 100%, respectively, of *Set1* KD testes exhibited this early-stage germ cell overpopulation phenotype (Fig. 1C,D). Here, we used a membrane marker (anti-Armadillo) to identify cyst cells that encapsulate germ cells, as shown previously (Feng et al., 2017). Any cyst with more than 16 germ cells was considered overpopulated. To test further whether these overpopulated germ cells resulted from over-proliferation, immunostaining using an antibody against a mitosis-enriched marker, H3S10P (phosphorylation at Serine 10 of H3) (Ranjan et al., 2022; Xie et al., 2015), revealed increased H3S10P-positive early-stage germ cells in the *Set1* KD testes at days 3, 5 and 7, compared with earlier time points (days 1 and 3) as well as the Ctrl KD testes throughout this time course (Fig. S2).

In addition to the germline phenotypes observed in the *Set1* KD testes, two other phenotypes were detected in the somatic cell lineages of the testis. First, the hub area was significantly increased compared with the Ctrl KD testes throughout this time-course experiment, with a higher degree of difference toward the later time points (Fig. 1F). Second, the number of cyst cells, positively stained with both a cyst cell marker, Traffic jam (Tj) (Li et al., 2003), and an early-stage cyst cell marker, Zinc-finger homeodomain protein 1 (Zfh1) (Eun et al., 2014; Issigonis et al., 2009; Leatherman and Dinardo, 2008), was significantly increased in the *Set1* KD testes compared with the Ctrl KD testes at the later time points in the time-course experiments (Fig. 1G, Fig. S3). These changes in cyst cell number coincided with the changes in the germline phenotypes over the duration of the time course in the germline *Set1* KD testes, indicating that Set1 acts in the germ cells to regulate somatic gonadal cells in a non-cell-autonomous manner.

**Fig. 2.**
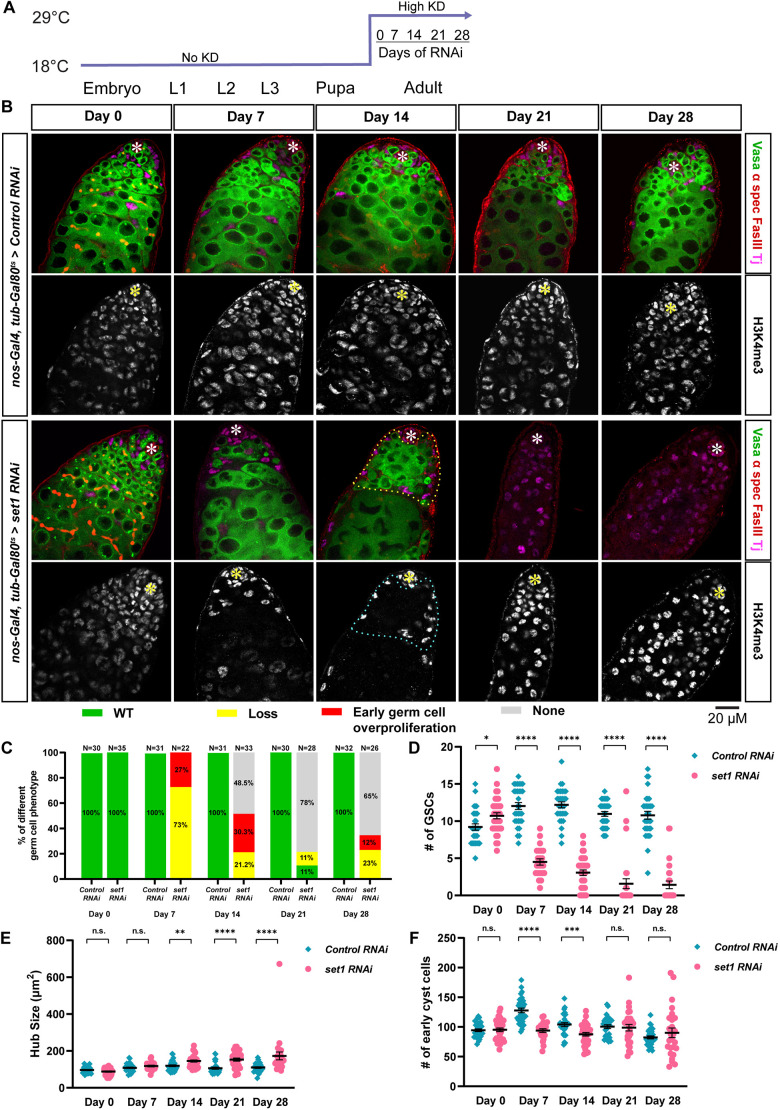
**Knockdown of *Set1* exclusively in the adult *Drosophila* testis leads to germ cell loss and germline differentiation defects.** (A) *tub-Gal80^ts^, nos>Control RNAi* and *tub-Gal80^ts^, nos>set1 RNAi* flies were grown at the permissive temperature (18°C) until eclosion, then shifted to the restrictive temperature (29°C) for 0, 7, 14, 21 or 28 days. (B) Representative images of *tub-Gal80^ts^, nos>Control RNAi* and *tub-Gal80^ts^, nos>set1 RNAi* testes at day 0, 7, 14, 21 and 28 post-eclosion. *vasa-GFP* from a knock-in strain label the germline, immunostaining for H3K4me3 (gray), Arm (red) or Fas III (red) labels the hub region, α-spectrin (red) the spectrosome and fusome, and Tj (magenta) the CySC lineage cells. Asterisks indicate the hub. Yellow or cyan dotted outlines indicate overpopulated early-stage germ cells. Notably, for day 21 and 28 *Set1* RNAi samples, no Vasa-positive cells could be detected, with all remaining cells being Tj-positive cyst cells. (C,D) Quantification of the percentage of testes with the germline phenotypes (C) and GSC number (D) in *tub-Gal80^ts^*, *nos>Control RNAi* testes (day 0: *n*=30; day 7: *n*=31; day 14: *n*=31; day 21: *n*=30; day 28: *n*=32) and *tub-Gal80^ts^*, *nos>set1 RNA*i testes (day 0: *n*=35; day 7: *n*=22; day 14: *n*=33; day 21: *n*=28; day 28: *n*=26). See also Table S1. (E) Quantification of hub region size for *tub-Gal80^ts^, nos>Control RNAi* and *tub-Gal80^ts^, nos>set1 RNAi* testes. See also Table S2. (F) Quantification of early cyst cell number for *tub-Gal80^ts^, nos>Control RNAi* and *tub-Gal80^ts^, nos>set1 RNAi* testes. See also Table S3. Individual data points and mean values are shown. Error bars represent s.e.m. ****P<10^−4^, ***P<10^−3^, **P<10^−2^, *P<0.05. n.s., not significant. Unpaired *t*-test was used to compare two individual datasets with each other.

Because the Gal4/UAS system may still be functional at 18°C, knockdown of Set1 at earlier stages of development could contribute to those phenotypes detected in adulthood (Brand and Perrimon, 1993). In *Set1* KD and Ctrl KD at L3, the overall early germline morphology in *Set1* KD testes resembled that of the Ctrl KD testes with no early-stage germ cell proliferation and less severe germ cell loss compared with adult *Set1* KD testes (Fig. 1C,D). Phenotypes such as germline stem cell number and hub size showed much less or no significant difference between the *Set1* KD and Ctrl KD testes, respectively (Fig. 1E,F), whereas the cyst cell number continued to increase in the *Set1* KD testes compared with the Ctrl KD testes from L3 to day 7 (Fig. 1G). Therefore, to probe further the requirement of Set1 in adulthood, we knocked down *Set1* in the early germline using the temperature-sensitive Gal80 controlled by the *tubulin* promoter (*tub-Gal80^ts^*). At the permissive temperature (18°C), functional Gal80 protein inhibits Gal4 from associating with the UAS sequences, thus turning on the RNAi expression, but at the restrictive temperature (29°C) Gal80 is inactivated and Gal4 can associate with UAS and activate RNAi (McGuire et al., 2003). The *tub-Gal80^ts^, nos>set1 RNAi* (*Set1* ts-KD) and *tub-Gal80^ts^, nos>Control RNAi* (Ctrl ts-KD) flies were grown at 18°C until eclosion, when they were shifted to 29°C for 0, 7, 14, 21 and 28 days, respectively, for another time-course experiment (Fig. 2A). At day 0, the *Set1* ts-KD and Ctrl ts-KD testes had comparable germline morphology, indicating that active Gal80 at 18°C effectively prevents *Set1* knockdown (Fig. 2B,C, day 0). However, 7 days after shifting to 29°C, 73% of the *Set1* KD testes showed germline loss, and 27% exhibited the early germline overpopulation phenotype (Fig. 2B,C, day 7). These results indicate that the early germline phenotypes can be induced upon knocking down *Set1* in adulthood. In addition to the germline phenotypes detected in the *Set1* KD time course (Fig. 1C-E), in *Set1* ts-KD testes at later time points, a new phenotype classified as ‘none’ was detected (no Vasa-positive germ cells could be detected at day 21 and day 28; Fig. 2B), whereby the testes are almost completely devoid of germ cells (Fig. 2B,C: 48.5% at day 14, 78% at day 21 and 65% at day 28). Throughout this time course, GSC number was consistently reduced at every time point in *Set1* ts-KD testes compared with the Ctrl ts-KD testes (Fig. 2D, days 7, 14, 21 and 28). These results demonstrate that Set1 is required intrinsically for GSC maintenance in adulthood. Furthermore, the hub area was significantly increased at the later time points in the *Set1* ts-KD testes (Fig. 2E, days 14, 21 and 28), whereas the cyst cell number was significantly reduced in *Set1* ts-KD testes at the earlier time points (Fig. 2F, day 7 and day 14). The variation in somatic gonadal cell phenotypes between *Set1* KD and *Set1* ts-KD could be due to the non-cell-autonomous effects induced by the strength of germline *Set1* KD and/or the different temperature shift regimes, as it has been shown that temperature contributes to germ cell differentiation in *Drosophila* (Gandara and Drummond-Barbosa, 2022, 2023). Together, both strategies to knock down *Set1* in the germline shown in Figs 1 and 2 demonstrate that Set1 is likely required cell-autonomously for adult GSC maintenance and proper differentiation.

### Set1 is not required in somatic gonadal cells or late-stage spermatogonial cells for the GSC loss phenotype

To determine whether the detected GSC loss phenotype (Figs 1E and 2D) is specific to *Set1* knockdown in the early germline, we first used cell type-specific strategies to knock down *Set1* in the CySC lineage using the *tj-Gal4* driver (Li et al., 2003). In *tj>set1 RNAi* testes, H3K4me3 signal is reduced in the CySCs and cyst cells compared with the signals in other cell types in the adult testis (Fig. S4A). At 7 days post eclosion, no obvious germline phenotype could be detected in the *tj>set1 RNAi* testes compared with the *tj>Control RNAi* testes (Fig. S5A). The number of GSCs was actually increased in the *tj>set1 RNAi* testes (Fig. S5D), contrary to what was detected in the *nos>set1 RNAi* testes (Figs 1E and 2D). The hub area exhibited no significant difference between the *tj>set1 RNAi* testes and the *tj>Control RNAi* testes (Fig. S4A′), but the number of cyst cells was significantly increased in the *tj>set1 RNAi* testes (Fig. S4A″). In addition, to determine the stage specificity of the GSC loss phenotype in *nos>set1 RNAi* testes, we used the stage-specific *bam-Gal4* (*bag of marbles*) driver, which turns on target gene expression specifically in 4- to 16-cell spermatogonial cysts (Chen and McKearin, 2003). In *bam>set1 RNAi* testes, H3K4me3 was diminished in the germline past the 4-cell spermatogonial cyst stage but still present in the GSCs and very early-stage germ cells (Fig. S4B). At 7 days post-eclosion, there were no obvious morphological changes in the germline of *bam>set1 RNAi* testes compared with *bam>Control RNAi* testes (Fig. S5B). In addition, the number of GSCs and the hub area in *bam>set1 RNAi* testes and *bam>Control RNAi* testes were not significantly different from each other (Fig. S5E), even though the *bam>set1 RNAi* testes had significantly more cyst cells (Fig. S4B-B″). In summary, these results indicate that knockdown of *Set1* in early-stage germ cells is responsible for the detected GSC loss phenotypes shown in Figs 1 and 2.

To confirm these findings, we used *nos-Gal4ΔVP16, bam-Gal80* to drive *Set1* RNAi only in the GSCs, gonialblasts, and 2-cell spermatogonial cysts as Gal80 protein driven by the *bam* promoter prevents RNAi expression in 4- to 16-cell spermatogonial cysts (Eliason et al., 2018). In the *nos-Gal4ΔVP16, bam-Gal80>set1 RNAi* testes, 95% had a reduction in H3K4me3 specifically in GSCs, gonialblasts and early-stage spermatogonial cells (Fig. S4C). At 7 days post-eclosion, the hub size and number of cyst cells in the *nos-Gal4ΔVP16, bam-Gal80>set1 RNAi* testes were comparable to those in *nos-Gal4ΔVP16, bam-Gal80>Control RNAi* testes (Fig. S4C′,C″). However, the number of GSCs in the *nos-Gal4ΔVP16, bam-Gal80>set1 RNAi* testes was significantly reduced compared with the *nos-Gal4ΔVP16, bam-Gal80>Control RNAi* testes (Fig. S5F). Together, these data demonstrate that knockdown of *Set1* in the early-stage germ cells can partially account for the GSC loss phenotype seen in the *nos>set1 RNAi* testes. The differences in other phenotypes, especially the non-cell-autonomous phenotypes, are likely due to the cell type and stage specificities, as well as the strength of the KD effects, using different drivers. We focused on using *nos>set1 RNAi* testes for the following experiments to understand the mechanisms underlying the *Set1* KD germline phenotypes.

### The methyltransferase activity of Set1 is required for its proper activity in germ cells

**Fig. 3.**
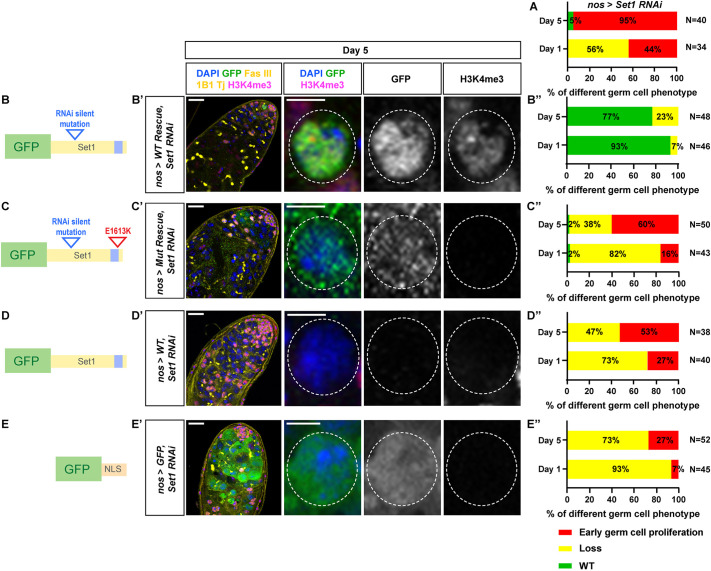
**The methyltransferase activity of Set1 is required for its function in the male germline.** (A) Quantification of the percentage of testes with the representative germline phenotypes, germ cell loss and early-germline overpopulation, present in *nos>set1 RNAi* testes at 1 day and 5 days post-eclosion (adapted from Fig. 1D). (B-E) Schematics of the *Set1* cDNA transgene with the RNAi recognition sequences mutated, named WT rescue (B); the *Set1* cDNA transgene with the RNAi recognition sequences mutated and an E→K amino acid change in the SET domain, named Mut rescue (C); the *Set1* cDNA transgene without the RNAi recognition sequences mutated, named WT (D); the *GFP* cDNA with the nuclear localization sequence, named GFP (E). (B′-E′) Representative images of *nos>WT Rescue, set1 RNAi* testis (B′), *nos>Mut Rescue, set1 RNAi* testis (C′), *nos>WT, set1 RNAi* testis (D′) and *nos>GFP, set1 RNAi* testis (E′). All testes are from males 5 days post-eclosion, immunostained with H3K4me3 (magenta), GFP (green), Fas III (yellow) for the hub region, 1B1 (yellow) for spectrosome and fusome, and Tj (yellow) for the CySC lineage cells. Right-hand panels show higher magnification images of an example germ cell expressing the corresponding transgenes (outlined by white dashed lines). Scale bars: 20 μm (testis); 5 μm (individual germ cell images). (B″-E″) Quantification of the percentage of testes with the representative germline phenotypes, germ cell loss and early-germline overpopulation, present in *nos>WT Rescue, set1 RNAi* testes (B″), *nos>Mut Rescue, set1 RNAi* testes (C″), *nos>WT, set1 RNAi* testes (D″), *nos>GFP, set1 RNAi* testes (E″), at 1 and 5 days post-eclosion.

To confirm that the phenotypic effects seen in the *nos>set1 RNAi* testes (Fig. 3A, adapted from Fig. 1D) are due to the knockdown of the *Set1* gene, but not off-targets effects of RNAi, a rescue experiment was performed. In *nos>set1 RNAi* testes, a *GFP*-tagged *Set1* cDNA transgene was expressed using the same *nos-Gal4* driver. Furthermore, because the *Set1 RNAi* target sequence is within the coding region of *Set1*, silent mutations were made within the target region to prevent KD of the transgene, namely a GFP-tagged *Set1* cDNA transgene with the RNAi silent mutations (*WT Rescue*; Fig. 3B, Fig. S6A). As controls, three additional transgenes were generated and crossed individually to the same *Set1* RNAi background to perform rescuing experiments: the *WT Rescue* transgene with RNAi silent mutations and an E-to-K mutation in the catalytic SET domain (*Mut Rescue*; Fig. 3C, Fig. S6B), a GFP-tagged *Set1* cDNA transgene without the RNAi silent mutations (*WT*; Fig. 3D, Fig. S6C), and a GFP transgene with a nuclear localization signal (*GFP-NLS*; Fig. 3E, Fig. S6D). At day 1 and day 5 post eclosion, 93% and 77% of the *nos>WT Rescue, set1 RNAi* testes showed full rescue of the *Set1* knockdown phenotypes (Fig. 3B′,B″, Fig. S6A′). By contrast, in the *nos>Mut Rescue, set1 RNAi* testes, only 2% showed full rescue, whereas 82% and 38% exhibited the germline loss phenotype, as well as 16% and 60% displaying the early germ cell proliferation phenotype at day 1 and day 5 post eclosion, respectively (Fig. 3C′,C″, Fig. S6B′), indicating that the catalytic SET domain is indispensable for the normal function of Set1 in the early-stage male germline. Both the *nos>WT, set1 RNAi* testes and *nos>GFP-NLS, set1 RNAi* testes show similar results compared with the *nos>Mut Rescue, set1 RNAi* testes (Fig. 3D′,D″,E′,E″, Fig. S6C′,D′), suggesting that adding back *Set1* without changing the RNAi recognition sequences or a non-specific GFP-NLS transgene does not rescue the *Set1* knockdown phenotypes in the male germline.

Furthermore, the GFP signals from both the *nos>WT Rescue* and the *nos>Mut Rescue* GFP-tagged fusion proteins are exclusively detected in the early germline nuclei, indicating that the silent mutations make these transgenes resistant to the RNAi knockdown, allowing for their proper expression and localization (Fig. 3B′,C′, Fig. S6A′,B′). In contrast, no GFP signal could be detected in the *nos>WT, set1 RNAi* testes without the RNAi silent mutations, indicating effective knockdown. In summary, these results support that the germline phenotypes observed in *nos>set1 RNAi* testes are due to the methyltransferase activity of the Set1 protein.

### *Set1* regulates expression of multiple signaling pathway components in the *Drosophila* testis

**Fig. 4.**
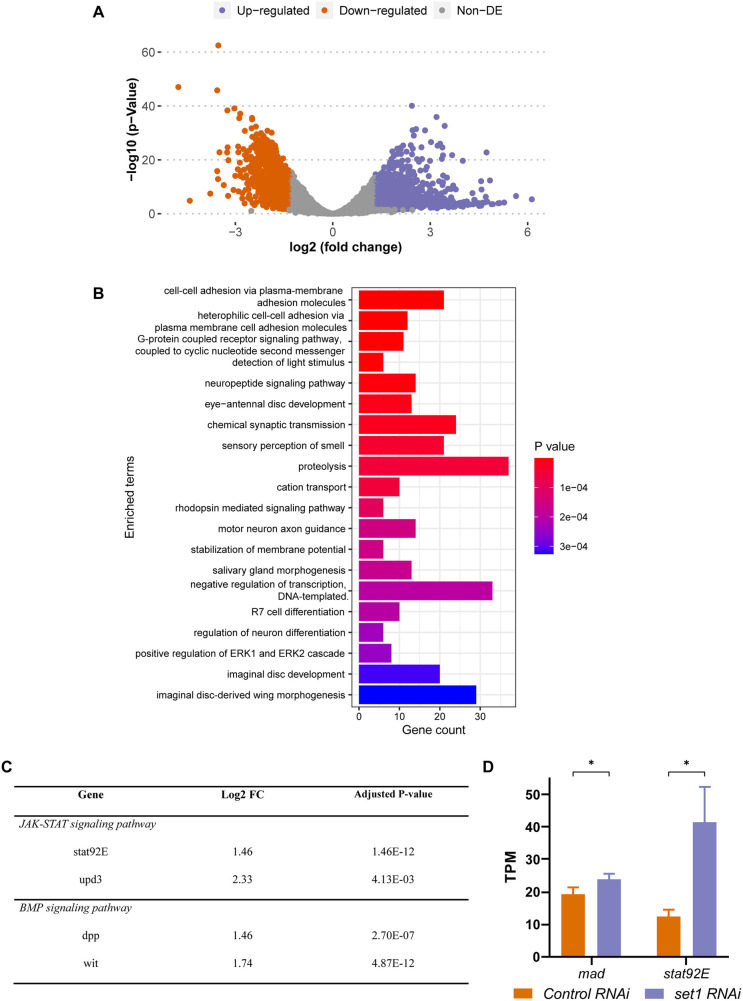
**JAK-STAT and BMP signaling pathway components have increased expression in *Set1* knockdown testis.** (A) Volcano plot of differentially expressed genes in *Set1* KD testes versus Ctrl KD testes at 3 days post-eclosion (≥log_2_1.3=2.46-fold change, *P*<0.05). Non-DE, not differentially expressed. (B) GO analysis of the 1216 upregulated genes identified from A. GO analysis of the 764 downregulated genes did not show any significant category. (C) Several JAK-STAT and BMP signaling pathway components that are upregulated in *Set1* KD testes compared with Ctrl KD testes 3 days post-eclosion. (D) Expression levels of *Mad* and *Stat92E* in *Set1* KD testes versus Ctrl KD testes. Error bars represent s.e.m., *n*=3 biological replicates, *P<0.05 using unpaired *t*-test. TPM, transcripts per million reads.

To understand the molecular mechanisms underlying the function of Set1 in the germline, RNA sequencing (RNA-seq) was performed with three independent biological replicates to compare the transcriptomes between *nos>Control RNAi* (Ctrl KD) and *nos>set1 RNAi* (*Set1* KD) testes at 0, 1, 3 and 5 days post-eclosion, respectively (Fig. 4, Fig. S7). To obtain a global picture of the transcriptome changes between Ctrl KD and *Set1* KD testes at different time points, principal component analyses were performed for all 24 samples (Fig. S7A). Among the 24 samples, the Ctrl KD samples cluster together and the *Set1* KD samples cluster together (Fig. S7A). In addition, the biological replicates for each *Set1* KD sample time points cluster more closely with one another than the replicates of the Ctrl KD sample time points (Fig. S7A). This can be explained as all 12 Ctrl KD samples should have similar gene expression patterns to one another given that the age difference between each consecutive time points is only 1-2 days. In contrast, the 12 *Set1* KD samples do not, as visible changes could be detected at each time points in the *Set1* KD testes. To analyze in more detail the transcriptome changes between the Ctrl KD and *Set1* KD samples at every time point, a heatmap of all the differentially expressed genes was created (Fig. S7B). The 16,610 genes included in the heatmap, according to the *Drosophila* Release 6 reference genome (dos Santos et al., 2015), could be classified into four groups. Group 1 contains genes that are highly expressed at the earlier time points of the *Set1* KD samples (days 0 and 1), whereas Group 2 has the genes that are highly expressed at the later time points of the *Set1* KD samples (days 3 and 5). Additionally, Group 3 consists of the genes that are downregulated in the *Set1* KD samples. Finally, Group 4 comprises the genes that had variable expression profiles across all the time points and thus did not fit the clear expression patterns seen for the genes in groups 1-3 (Fig. S7B).

After these initial analyses on all 24 samples, we focused on day 3 of the *Set1* KD samples for two reasons. First, it is the time point when the germline mass phenotype became predominant; second, it had the most differentially expressed genes even with a stringent cutoff (≥log_2_1.3=2.46-fold and *P*<0.05; Fig. 4A, Fig. S7C). At this time point, 1216 genes were significantly upregulated and 764 genes were significantly downregulated in the *Set1* KD samples compared with the Ctrl KD samples (Fig. 4A, Fig. S7C). Gene ontology (GO) analysis of the 1216 upregulated genes showed enriched functions, with a significant category being ‘negative regulation of transcription’ (Fig. 4B).

Interestingly, components of multiple signaling pathways, such as JAK-STAT, BMP, EGF, Notch, Hedgehog, Wnt and Hippo signaling pathways, were found to be upregulated in the *Set1* KD compared with the Ctrl KD samples at day 3 (Fig. S7D). For the EGF, Hedgehog and Wnt signaling pathways, multiple core components were significantly upregulated, whereas for the Notch and Hippo pathways some components were significantly upregulated and others were significantly downregulated. Among all these, only the JAK-STAT, BMP and EGF signaling pathways had at least two core components that made the significant cut-offs (Fig. 4C, Fig. S7D). For example, the JAK-STAT pathway genes *Stat92E* and *upd3*, and the BMP pathway genes *dpp* and *wit*, were found to be significantly upregulated (Fig. 4C). Given the importance of both signaling pathways in the testis (Inaba et al., 2015; Kawase et al., 2004; Kiger et al., 2001; Leatherman and Dinardo, 2008, 2010; Schulz et al., 2004; Singh et al., 2010; Tulina and Matunis, 2001), these results indicate that the early-stage germ cell overpopulation phenotype could be due to ectopic expression of both JAK-STAT and BMP signaling pathway genes.

### The interactions between *Set1* and JAK-STAT or BMP pathway genes are likely responsible for the early-stage germline phenotypes

To investigate further the functional relevance of both JAK-STAT and BMP signaling pathways with the *Set1* KD phenotypes, we examined two key downstream genes of JAK-STAT and BMP pathways, *Stat92E* and *Mad*, respectively. Both genes were identified to be upregulated in the *Set1* KD testes compared with the Ctrl KD testes based on the RNA-seq results (Fig. 4D). However, given that Set1 generates H3K4me3 to activate transcription, the direct target genes of Set1 are likely downregulated in *Set1* KD testes. Nevertheless, we reason that the ectopic expression of *Stat92E* or *Mad* could contribute to the early-germline overpopulation phenotypes in the *Set1* KD testes. Next, we tested potential genetic interactions of them with *Set1*.

**Fig. 5.**
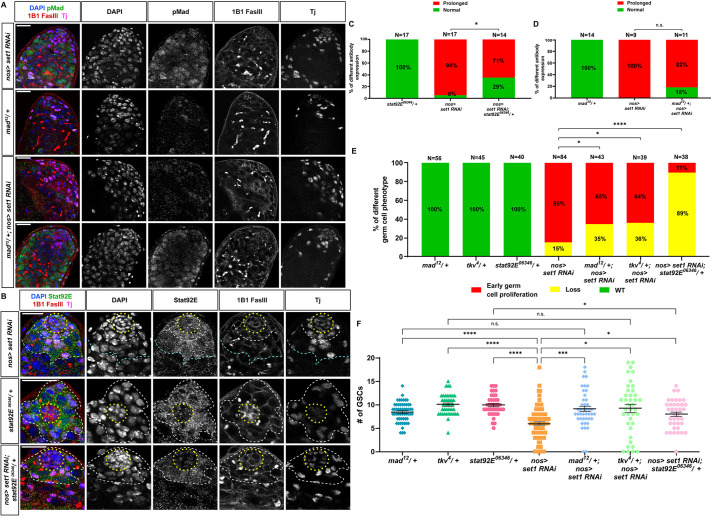
**Set1 regulates key JAK-STAT and BMP signaling pathway components.** (A) Representative images of *nos>set1 RNAi*, *mad^12^/+* and *nos-Gal4/mad^12^* (on the 2nd chromosome, abbreviated as *mad^12^/+*)*; nos>set1 RNAi* testes at 5 days post-eclosion, immunostained for pMad (green), Fas III (red) for the hub region, 1B1 (red) for spectrosome and fusome, and Tj (magenta) for the CySC lineage cells. (B) Representative images of *nos>set1 RNAi*, *stat92E^06346^/+* and *UAS-set1 RNAi*/*stat92E^06346^* (on the 3rd chromosome, abbreviated as *stat92E^06346^/+*)*; nos>set1 RNAi* testes at 5 days post-eclosion, immunostained for Stat92E (green), Fas III (red) for the hub region, 1B1 (red) for spectrosome and fusome, and Tj (magenta) for the CySC lineage cells. In B, yellow dotted outline delineates the hub, white dashed outline the GSCs and CySCs region, and cyan dashed outline the spermatogonial region. Scale bars: 20 μm. (C) Quantification of the percentage of testes with representative Stat92E antibody expression: normal or ectopic with prolonged expression patterns present in *stat92E^06346^/+* versus *nos>set1 RNAi* versus *nos>set1 RNAi; stat92E^06346^/+* testes at 5 days post-eclosion. *P<0.05, χ^2^ test. (D) Quantification of the percentage of testes with representative pMad antibody expression: normal or ectopic with prolonged expression patterns present in *mad^12^/+* versus *nos>set1 RNAi* versus *mad^12^/+; nos>set1 RNAi* testes at 5 days post-eclosion*.* n.s., not significant, χ^2^ test. (E) Quantification of the percentage of testes with the representative germline phenotypes, germ cell loss and early-germline overpopulation, using the same criteria as defined in Fig. 1D, present in *mad^12^/+* (*n*=56), *tkv^4^/+* (*n*=45), *stat92E^06346^/+* (*n*=40), *nos-Gal4>set1 RNAi* (*n*=84), *mad^12^/+; nos-Gal4>set1 RNAi* (*n*=43)*, tkv^4^/+; nos-Gal4>set1 RNAi* (*n*=39) and *stat92E^06346^/+; nos-Gal4>set1 RNAi* (*n*=38) testes at 5 days post-eclosion. *P<0.05, ****P<10^−4^, χ^2^ test. (F) Quantification of GSC number for *mad^12^/+* (*n*=56), *tkv^4^/+* (*n*=45), *stat92E^06346^/+* (*n*=40), *nos-Gal4>set1 RNAi* (*n*=84), *mad^12^/+; nos-Gal4>set1 RNAi* (*n*=43)*, tkv^4^/+; nos-Gal4>set1 RNAi* (*n*=39) and *stat92E^06346^/+; nos-Gal4>set1 RNAi* (*n*=38) testes at 5 days post-eclosion. Individual data points and mean values are shown. See also Table S1. Error bars represent s.e.m. *P<0.05, ***P<10^−3^, ****P<10^−4^; n.s., not significant; one-way ANOVA and Dunnett's T3 multiple comparison test was used to compare multiple individual datasets with each other that had one independent variable.

In heterozygous loss-of-function *mad^12^* testes (Raftery et al., 1995) or Ctrl KD testes, pMad was detectable in the GSCs (Fig. 5A,D, *mad^12^/+*; Fig. S8A) and 100% showed normal morphology (Fig. 5E). In contrast, in the *Set1* KD testes, pMad was detected in GSCs and ectopically in the spermatogonial cells (Fig. 5A,D, *nos>set1 RNAi*). Overall, 85% of the *Set1* KD testes exhibited the early germ cell overpopulation phenotype (Fig. 5E). Even though both pMad patterns were detectable in *Set1* KD testes with one copy of the *mad^12^* allele (Fig. 5A,D, *mad^12^/+; nos>set1 RNAi*), the addition of one *mad^12^* allele to the *Set1* KD background reduced the occurrence of the early germ cell overpopulation phenotype from 85% to 65% (*P*<0.05; Fig. 5E). Furthermore, we tested *thickveins* (*tkv*), which encodes an upstream receptor of the BMP pathway and displays increased expression in the *Set1* KD testes compared with the Ctrl KD testes (Fig. S7D). The loss-of-function *tkv^4^* allele (Lecuit and Cohen, 1997; Terracol and Lengyel, 1994) also suppressed the early germ cell overpopulation phenotype from 85% to 64% (*P*<0.05; Fig. 5E), as well as the GSC loss phenotype (*P*<0.05; Fig. 5F). Together, these results suggest that Set1 normally represses the BMP signaling pathway. Without this repression, ectopic activity can occur, leading to abnormal germline morphology and composition.

Additionally, in heterozygous testes with the loss-of-function *stat92E^06346^* allele (Hou et al., 1996) or Ctrl KD testes, Stat92E expression was detected in the GSCs (white outline in Fig. 5B,C, *stat92E^06346^/+*; Fig. S8B), but in *Set1* KD testes Stat92E was not only detectable in GSCs but also in hub cells (yellow outline in Fig. 5B, *nos>set1 RNAi*) and in spermatogonial cells (cyan outline in Fig. 5B, *nos>set1 RNAi*). Interestingly, upon compromising Stat92E with *stat92E^06346^* in the *Set1* KD background, the expression of Stat92E was no longer found in hub cells (yellow outline in Fig. 5B, *nos>set1 RNAi; stat92E^06346^/+*) or in the spermatogonial cells. Furthermore, when the level of Stat was reduced in *nos>set1 RNAi; stat92E^06346^/+* testes, the incidence of the early-stage germ cell overpopulation phenotype decreased from 85% to 11% (*P*<10^−4^; Fig. 5E). These results suggest that enhanced Stat92E expression upon Set1 inactivation significantly contributes to the early germ cell overpopulation phenotype in *Set1* KD testes. Moreover, compromising the BMP pathway by *mad^12^/+* fully suppresses the GSC loss phenotypes in *Set1* KD testes, whereas compromising the BMP pathway by *tkv^4^* can also partially suppress the GSC loss phenotype in *Set1* KD testes (*P*<0.05; Fig. 5F). Halving the level of Stat92E could partially suppress the GSC loss phenotype in *Set1* KD testes (*P*<0.05; Fig. 5F). However, none of the conditions could fully restore the loss of some spermatogonial cells in the *Set1* KD testes (Fig. 5E), suggesting that other downstream factors of these two signaling pathways or components from other potentially involved signaling pathways (Fig. S7D) could contribute to the loss of early-stage germ cells at the *Set1* KD background. Together, these genetic interaction results support the functional relationships between *Set1* and BMP as well as JAK-STAT pathways, with the primary role of BMP signaling relating to the GSC loss phenotype and a predominant contribution of the JAK-STAT pathway to the early-stage germline overpopulation phenotype, consistent with the transcriptome results shown in Fig. 4.

## DISCUSSION

**Fig. 6.**
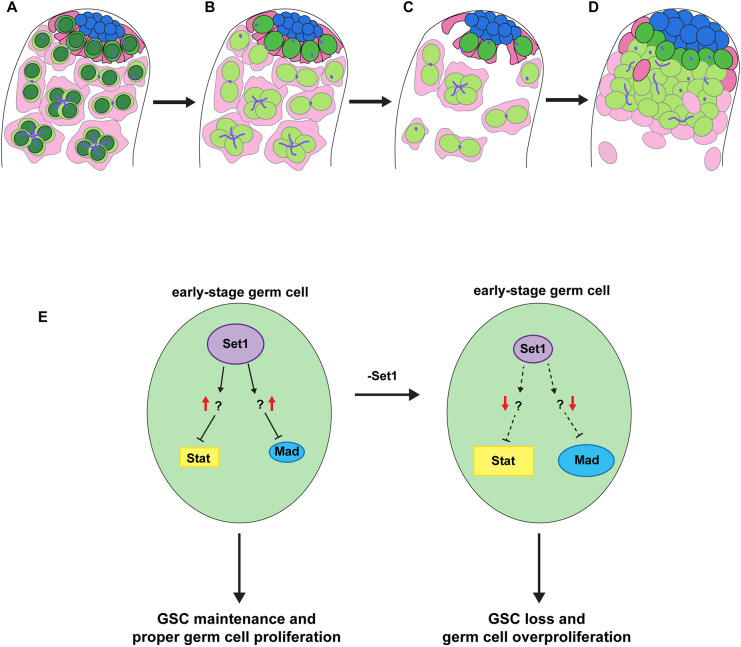
**Illustration of the progression of *Set1* knockdown phenotypes in adult testis.** (A) In a wild-type testis, H3K4me3 is present in all cell types of the testis, but for simplicity the presence of H3K4me3 (dark green) is only shown in the germ cells, including GSCs (bright green), gonialblasts and spermatogonia (light green). CySCs are shown in magenta, cyst cells in pink, and hub cells in blue. (B) Knockdown of *Set1* in the early germline results in a reduction of H3K4me3 in GSCs, gonialblasts and spermatogonia. (C) The phenotypes at the earlier time points include a reduction of germ cells and cyst cells. (D) The phenotypes at the later time points include overpopulated early-stage germ cells ectopically expressing Stat92E and Mad, as well as increased hub size. (E) Putative pathways of Set1 function in the early-stage germline, including GSCs. Given that both *Stat92E* and *Mad* have increased expression in the *Set1* KD testes, they are likely indirect targets of Set1. One possible explanation is that Set1 directly regulates inhibitors of both *Stat92E* and *Mad* (left). Compromising *Set1* could lead to the downregulation of these inhibitors and ectopic expression of *Stat92E* and *Mad*, resulting in the GSC loss and early-stage germ cell overproliferation phenotypes (right).

This study explores the *in vivo* roles of *Drosophila* Set1, a histone methyltransferase responsible for ‘writing’ the H3K4me3 histone modification, in the *Drosophila* testis. Through a series of cell type- and stage-specific RNAi knockdown via a time course regime, our findings unveil a fascinating progression of germline defects (Fig. 6A-D). These *Set1* loss-of-function phenotypes have both cellular and molecular specificities. First, the phenotypes are predominantly linked to the loss of Set1 function in early-stage germ cells, as compromising Set1 in late-stage germ cells or somatic gonadal cells fails to replicate these defects. Moreover, we conducted rescuing experiments utilizing transgenes encoding both the wild-type and catalytic inactive forms of Set1. Only the wild-type Set1 effectively rescues the knockdown phenotypes, underscoring the specificity of these effects to the methyltransferase activity of Set1. To further understand the underlying molecular mechanisms, we performed RNA-seq experiments to identify genes with changed expression upon knocking down *Set1 g*ene. Through this assay, we identified several crucial signaling pathway genes, such as *Stat92E* and *Mad*, the downstream components of the JAK-STAT and BMP signaling pathways, respectively, that have increased expression when *Set1* is knocked down. Genetic interaction analyses further suggest a functional relationship between *Set1* and these signaling pathways, as mutations of the *Stat92E* and *Mad* genes suppress the *Set1* knockdown phenotypes. Collectively, our investigations shed light on the molecular mechanisms at play, enhancing our comprehension of the pivotal decision-making process governing proliferation versus differentiation within adult stem cell lineages. This insight bears significant implications for both stem cell biology and the broader field of cancer biology.

### Set1 may regulate JAK-STAT and BMP signaling pathway inhibitors in the *Drosophila* male germline

Here, our RNA-seq results reveal that upon knockdown of *Set1* in the *Drosophila* male germline, a key downstream factor of the JAK-STAT signaling pathway, *Stat92E*, is upregulated (Fig. 4D). This is unexpected for the direct targets of Set1, given that Set1 is the methyltransferase for the transcriptional activating mark H3K4me3. In addition, previous ChIP-seq results using progenitor germ cell-enriched *bam* testes (McKearin and Spradling, 1990) indicate that H3K4me3 is not enriched at the promoter region of the *Stat92E* gene (Fig. S9A) (Gan et al., 2010). These results suggest that *Stat92E* is unlikely to be a direct target of Set1. However, given the suppression of the early germ cell overpopulation phenotype when adding a single copy of a *Stat92E* mutation in the *Set1* KD background, they likely interact *in vivo*. We hypothesize that Set1 acts in the *Drosophila* male germline by regulating the transcriptional activity of a JAK-STAT inhibitor. When Set1 is compromised, the expression of this inhibitor is possibly downregulated, allowing for the ectopic expression of Stat92E (Fig. 6E). One candidate JAK-STAT inhibitor is protein tyrosine phosphatase 61F (Ptp61F). Ptp61F is a known inhibitor of the JAK-STAT pathway that has been previously identified as a target of Lid, the H3K4 demethylase, in the germline (Baeg et al., 2005; Tarayrah et al., 2015). Additionally, *Ptp61F* is slightly downregulated in the RNA-seq data (Fig. S9G) and is enriched with the H3K4me3 mark at its promoter regions (Fig. S9C).

In addition to Stat92E, the downstream factor of the BMP signaling pathway *Mad* is also upregulated in the *Set1* KD background (Fig. 4D). Unlike *Stat92E*, H3K4me3 is enriched at the promoter region of the *Mad* gene in progenitor germ cells (Fig. S9B) (Gan et al., 2010). However, given that *Mad* is slightly upregulated (Fig. 4D) and suppression of the early germ cell overpopulation phenotype was detected when a single copy of *Mad* mutation is combined with the *Set1* KD condition (Fig. 5A,E), we hypothesize that BMP inhibitor-encoding genes could be direct targets of Set1, downregulation of which upon inactivation of Set1 could lead to the ultimate upregulation of *Mad*. When examining the candidate BMP inhibitors Cul-2, MAN1 and Ube3a (Cul-2 is a BMP inhibitor in the *Drosophila* female germline, whereas both MAN1 and Ub3a are BMP inhibitors in other *Drosophila* tissues, as shown previously; Ayyub et al., 2015; Duan et al., 2020; Laugks et al., 2017; Li et al., 2016; Wagner et al., 2010), all three BMP inhibitors have downregulated expression to varying degrees in the *Set1* KD testes (Fig. S9G) and H3K4me3 enrichment can be detected at their corresponding promoter regions (Fig. S9D-F) (Gan et al., 2010). Additional experiments are needed to determine whether these inhibitors of JAK-STAT and BMP signaling are bona fide direct target genes and have a functional relationship with Set1 in the *Drosophila* male germline.

### Set1 may regulate other signaling pathways in the *Drosophila* male germline

Notch signaling has been shown to regulate the transit-amplification stage (Ng et al., 2019). Removal of *Notch* in the cyst cells or *Delta* in the germline result in spermatogonial cell death. Although *Delta* is not differentially expressed, *Notch* is upregulated whereas two other Notch pathway components, *Suppressor of Hairless* [*Su(H)*] and *Serrate* (*Ser*), have decreased expression in *Set1* KD testes (Fig. S7D). Increased expression of Wnt signaling components, such as *arm*, is detected using RNA-seq (Fig. S7D), which could result in abnormal clusters enriched with undifferentiated germ cell as reported (Pancratov et al., 2013). Finally, the EGF signaling is important for cyst cell differentiation and germline maintenance and differentiation (Feng et al., 2017), which regulates encapsulation of germ cells by the cyst cells (Amoyel et al., 2016; Schulz et al., 2004; Tran et al., 2000). The increased expression of multiple EGF signaling components in *Set1* KD testes (Fig. S7D) could act in conjunction with JAK-STAT and BMP signaling pathways. Because our RNA-seq analyses used the entire tissue, further studies are needed to address all these possibilities.

### Crosstalk between Set1 and other histone-modifying enzymes in regulating key signaling pathways

In *Drosophila*, the *lid* gene encodes the histone demethylase responsible for ‘erasing’ the H3K4 methylation (Eissenberg et al., 2007; Lee et al., 2007b), enzymatic activity of which directly antagonizes the function of Set1. It has been shown that in the male germline Lid is required for the proper levels of Stat92E. In the *lid* knockdown or mutant cells, *Stat92E* expression is reduced (Tarayrah et al., 2015). Therefore, the two histone-modifying enzymes with opposite activities, Set1 and Lid, display antagonistic regulation of *Stat92E* expression. Therefore, this work introduces a new epigenetic regulator within the complex framework of the JAK-STAT signaling pathway in the *Drosophila* testis niche. Together, these studies highlight the delicate balance maintained by a histone methyltransferase and a histone demethylase in orchestrating a major signaling pathway.

### Common and distinct roles of *Drosophila* Set1 in various stem cell systems

As the primary H3K4 methyltransferase in *Drosophila*, Set1 has also been demonstrated to play crucial roles in the female germline. For example, it has been shown that Set1 acts with the E3 ubiquitin ligase Bre1 to produce the majority of H3K4me3 modifications in regulating the maintenance and differentiation of female GSCs. Knockdown of *Set1* in the early-stage germline results in a significant loss of GSCs in the germaria, due to decreased expression of BMP signaling components, such as Mad or Dad (Bray et al., 2005; Xuan et al., 2013). This phenotype resembles the male GSC loss phenotype observed in this study. However, in the male germline, inactivation of Set1 predominantly affects the JAK-STAT pathway over the BMP pathway in our study. This effect is observed to progress over time, resulting in upregulated Stat92E levels. Consequently, we observe an overpopulation of early-stage germ cells during the late phase of our time-course experiments. Based on an extensive RNAi screen that identified hundreds of candidate genes with potential roles in the female germline, *Set1* stands out as a crucial factor required for female GSC maintenance and proper differentiation. When *Set1* is knocked down in the female germline, ovaries exhibit a spectrum of phenotypes, including empty ovarioles likely due to female GSC loss as well as so-called ‘pseudoegg’ chambers filled with undifferentiated cells (Yan et al., 2014). The latter phenotype resembles the early-germline overpopulation exhibited in the *Set1* male germline knockdown effects shown in this work (Fig. 1C,D). Moreover, for the non-cell-autonomous roles of Set1 in the *Drosophila* ovary, Set1 acts in somatic gonadal cells to impact the female germline. For example, compromising Set1 in different somatic gonadal cells in the ovary results in either GSC loss or overpopulated GSC-like cells in the germaria (Xuan et al., 2013). By contrast, compromising *Set1* in the somatic gonadal cells in the testis does not seem to cause any obvious phenotypes (Fig. S4A). However, in the *Drosophila* testis Set1 acts in the germline non-cell autonomously to affect niche architecture and cyst cell number (Fig. 1F,G).

In the somatic stem cell lineages, two types of neuroblast (NBs), type I NBs (NB-I) and type II NBs (NB-II), differ in number (e.g. 184 NB-Is and 16 NB-IIs) and lineage progression to ultimately produce the diverse range of neurons and glia in the brain (Bello et al., 2008; Boone and Doe, 2008). Interestingly, Set1 seems to be dispensable for the NB-II differentiation, which is distinct from the profound functions of Set1 in both ovary and testis for proper GSC differentiation (Yan et al., 2014), suggesting different requirements of histone modifications and their modifying enzymes in distinct stem cell systems.

### The progression of germline phenotypes resulting from Set1 loss of function underscores the need for caution when considering histone methyltransferase inhibitors in cancer therapy

Many short-lived cell types in the body are produced from adult stem cells, either continuously or in response to physiological signals or trauma. During these processes, genetic lesions or epigenetic misregulations can drive cells to divide continuously instead of undergoing proper differentiation. This shift in cellular properties can initiate diseases such as cancer.

The COMPASS complex has been shown to be involved in pathogenesis such as cancer (Shilatifard, 2012). Here, our results demonstrate significant phenotypic changes resulting from germline knockdown of *Set1* in fly testes, beginning with germ cell loss and progressing to the development of germline tumors within a relatively short timeframe (Fig. 1B-D). This delayed appearance raises an intriguing possibility: a resilient subset of ‘survivor cells’, which evade early cell death, initiates a phase of uncontrolled and rapid over-proliferation of undifferentiated cells, reminiscent of the phenomenon observed for ‘cancer stem cells’ (Beck and Blanpain, 2013). Given that various histone methyltransferases are targeted for therapeutic treatment in cancers, our discovery of initial cell loss followed by over-proliferation in response to knockdown of a key histone methyltransferase raises concerns about the potential use of these enzyme inhibitors in cancer therapy (Morera et al., 2016).

## MATERIALS AND METHODS

### Fly strains and husbandry

Flies were raised under standard yeast/molasses medium at 25°C unless stated otherwise. The following flies were used: *nos-Gal4 (with VP16)/Cyo* (Van Doren et al., 1998), *nos-Gal4 (without VP16* or *ΔVP16)* on the second chromosome (from Yukiko Yamashita, University of Michigan, USA), *UAS-set1 RNAi* (Bloomington *Drosophila* Stock Center, BL33704), *UAS-mCherry RNAi* (Bloomington *Drosophila* Stock Center, BL35785), *tj-Gal4/Cyo* (Tanentzapf et al., 2007), *bam-Gal4/TM6B* (Chen and McKearin, 2003)*, bam-Gal80* on the third chromosome (from Juliette Mathieu and Jean-René Huynh, Collège de France, France), *UAS-GFP.nls* (Bloomington *Drosophila* Stock Center, BL4775), *UASp-FRT-EGFP-set1 (WT)-PolyA-FRT* (this study), *UASp-FRT-EGFP-set1 (WT rescue)-PolyA-FRT* (this study), *UASp-FRT-EGFP-set1 (Mutant rescue)-PolyA-FRT* (this study), *mad^12^, FRT40A/Cyo* (Bloomington *Drosophila* Stock Center, BL58785), *stat92E^06346^/TM3, Sb* (Bloomington *Drosophila* Stock Center, BL11681), *tkv^4^ FRT40A/Cyo* (Bloomington *Drosophila* Stock Center, BL58786), *vasa-GFP knock-in* (from Dr Tatjana Trcek, Johns Hopkins University, Baltimore, MD, USA).

### Spatiotemporally controlled experiments

To study the function of Set1 in the *Drosophila* testis, two RNAi lines, *UAS-set1 RNAi* and *UAS-mCherry RNAi*, were crossed with different drivers, *nos-Gal4/Cyo*, *bam-Gal4/TM6B* and *tj-Gal4/Cyo*, at 18°C. Flies at the larval stage L3 were collected and newly eclosed progenies were transferred to new vials at 25°C and aged for 0, 1, 3, 5 or 7 days before dissection.

For Set1 function study in the germline stem cells and gonialblast cells, the two RNAi lines *UAS-set1 RNAi* and *UAS-mCherry RNAi* were crossed with *nos-Gal4ΔVP16/Cyo; bam-Gal80/MKRS* at 25°C. Newly eclosed progeny were transferred to new vials and maintained at 25°C for 7 days before dissection.

To determine the role of Set1 in the *Drosophila* early germline at adulthood, two RNAi lines, *vasa-GFP knock-in/CyO; UAS-set1 RNAi* and *vasa-GFP knock-in/CyO; UAS-mCherry RNAi*, were crossed with *nos-Gal4/Cyo; tub-Gal80^ts^* at 18°C. Newly eclosed progenies were transferred to new vials at 29°C and aged for 0, 7, 14, 21 or 28 days before dissection.

To determine whether expression of *EGFP-set1* cDNA in the early germline is sufficient to rescue *nos>set1 RNAi* germ cell phenotypes, flies with the following genotypes were grown at 25°C and dissected at 1 and 5 days post eclosion: *nos-Gal4/UASp-FRT-EGFP-set1 (WT rescue)-PolyA-FRT*; *UAS-set1 RNAi/+*, *nos-Gal4/UASp-FRT-EGFP-set1 (Mutant rescue)-PolyA-FRT*; *UAS-set1 RNAi/+*, *nos-Gal4/UASp-FRT-EGFP-set1 (WT)-PolyA-FRT*; *UAS-set1 RNAi/+*, *nos-Gal4/UAS- GFP.nls*; *UAS-set1 RNAi/+*.

To identify whether *Set1* genetically interacts with *Mad*, *tkv* and *Stat92E*, the alleles *mad^12^*, *tkv^4^* and *stat92E^06346^* were used. Flies with the following genotypes were grown at 25°C and dissected at 5 days post eclosion: *nos-Gal4/mad^12^*; *UAS-set1 RNAi/+*, *nos-Gal4/mad^12^*, *nos-Gal4/tkv^4^*; *UAS-set1 RNAi/+*, *nos-Gal4/tkv^4^*, *nos-Gal4/+*; *UAS set1 RNAi/stat92E^06346^*, *UAS set1 RNAi/stat92E^06346^*; *nos-Gal4/+*; *UAS set1 RNAi/+*.

### Generation of transgenic fly lines

For the lines *UASp-FRT-EGFP-set1 (WT rescue)-PolyA-FRT* and *UASp-FRT-EGFP-set1 (Mutant rescue)-PolyA-FRT*, the RNAi recognition sequence was altered. Because the RNAi recognition sequence is in the coding region of the Set1 gene (AAGGTGCAGAGTATAAGAGTA), the third base for each codon of this sequence was changed to make a silent mutation that would allow for the protein to be translated properly but for the RNAi to not recognize the mRNA. For the line *UASp-FRT-EGFP-set1 (Mutant rescue)-PolyA-FRT*, a mutation in the SET domain at G4713A to make the E1613K amino acid replacement was made.

### Immunofluorescence

Testes were dissected in Schneider's insect media and then fixed in 4% formaldehyde in 1× PBST (1× PBS with 0.1% Triton X-100) for 8 min at room temperature (RT). The testes were rinsed three times in 1×PBST and washed three times for 5 min each time using 1× PBST at RT. Testes were incubated with primary antibodies in 1× PBST+3% bovine serum albumin at 4°C for at least one night. Samples were then rinsed three times in 1× PBST and washed three times for 5 min each time in 1× PBST and then incubated in a 1:1000 dilution of Alexa Fluor-conjugated secondary antibody in PBST+5% normal goat serum for 2 h at RT or rotating for a minimum of 24 h at 4°C. Samples were rinsed three times in 1× PBST and washed three times, 5 min each in 1× PBST and then mounted for microscopy in Vectashield antifade mounting medium (H-1400, Vector Laboratories) with or without DAPI. Samples were imaged using a Leica SP8 or Stellaris 5 confocal microscope with a 63× oil immersion objective. Images were analyzed using ImageJ software. Primary antibodies used were: Vasa (rabbit, 1:5000; from Ruth Lehmann, Skirball Institute of Biomolecular Medicine, NY, USA), Fas III (mouse, 1:50; DSHB, 7G10), Armadillo (mouse, 1:50; DSHB, N2 7A1), α-Spec (mouse, 1:50; DHSB, 3A9), 1B1 (mouse, 1:50; DSHB, 1B1-s), H3S10P (mouse, 1:2000; Abcam, ab14955), Zfh1 (rabbit, 1:5000; from Ruth Lehmann), TJ (guinea pig, 1:1000; from M. Van Doren, Johns Hopkins University, USA), H3K4me3 (rabbit, 1:400, Cell Signaling Technology, 9751S), GFP (chicken, 1:1000; Abcam, ab13970), Stat92E (rabbit, 1:200; gift from Denise Montell, University of Santa Barbara, CA, USA) and pMad (rabbit, 1:100; Abcam, ab52903). All secondary antibodies were Alexa Fluor Conjugated secondaries from Thermo Fisher Scientific used at 1:1000 dilution: goat anti mouse 405 A31553, 488 A23723, 568 A11004, 633 A21050, 647 A21235; goat anti rabbit 488 ab150077, 568 A11011, 633 A21202; goat anti guinea pig 488 A11073, 568 SAB4600080, 633 A21105; goat anti chicken 488 ab150169.

### RNA-seq and data analysis

*nos-Gal4; UAS-mCherry RNAi* and *nos-Gal4; UAS-set1 RNAi* flies were collected as newly eclosed males and aged for 0, 1, 3 and 5 days at 25°C after shift from 18°C. Approximately 15 pairs of testes for each genotype were dissected in Schneider's media+10% fetal bovine serum as one replicate. Three replicates were generated for each time point and genotype. The testes were then desheathed in 500 μl of lysis buffer (trypsin LE+2 mg/ml collagenase). The samples were incubated in lysis buffer for 10 min in a 37°C water bath with gentle vortex mixing every 2 min. The samples were then filtered through a 40 μm tissue culture filter followed by a 10 min centrifugation at 1200 rpm (106 ***g***). The cells were washed with 200 μl of PBS and pelleted again for 5 min at 1200 rpm (106 ***g***). Total RNA was purified using the Quick-RNA Microprep Kit (R1050, Zymo Research Corporation) following the manufacturer's instructions. The libraries were generated using the reagents provided in NEBNext Ultra II Directional RNA library Prep Kit for Illumina (E7760S, New England Biolabs Inc.) and NEB Next Poly(A) mRNA Magnetic Isolation Module (E7490, New England Biolabs Inc.). The Illumina compatible libraries were sequenced with Illumina Novaseq6000 sequencer at the National Institutes of Health sequencing facility.

The sequencing reads were examined using FastQC quality software (Galaxy Version 0.73+galaxy0) (https://www.bioinformatics.babraham.ac.uk/projects/fastqc/). Reads that passed the quality filter were then mapped to the *Drosophila* genome (D. melanogaster August 2014; BDGP Release 6+ISO11 MT/dm6) using Bowtie 2 version 2.5.1 (Langmead and Salzberg, 2012). For gene mapping, the gene model (Drosophila_melanogaster.BDGP6.87.gtf) was utilized, and we followed an RNA-seq data analysis tutorial using Galaxy (https://training.galaxyproject.org/training-material/topics/transcriptomics/tutorials/ref-based/tutorial.html), as detailed in the work (Batut et al., 2018; Hiltemann et al., 2023). Aligned reads were summarized to genes using featureCounts (Liao et al., 2014) with gene features obtained from NCBI RefSeq dm6 assembly. Raw counts were normalized to transcripts per million reads (TPM) counts. Principal component analysis was performed on 16,610 genes with detectable expression in at least one sample, based on which sample distance matrix was computed. To assess differential gene expression between Set1 knockdown and controls, DESeq2 (Love et al., 2014) was applied on samples from each day, using the Benjamini and Hochberg method for multiple testing correction with default DESeq2 parameters. A threshold of log_2_ fold change of 1.3 and adjusted *P*<0.05 was applied to ascertain differentially expressed genes, for which EnrichR (Chen et al., 2013) was applied to assess enrichment of molecular pathways in each set. All statistical analysis was conducted using R software version 4.2.0.

### Phenotype quantification

All phenotypic quantification was carried out in Fiji (ImageJ). GSC number was determined by counting every cell that was either Vasa positive or Tj negative and directly next to the hub. Early cyst cell number was quantified by counting every Tj-positive cell in a whole testis *z*-stack. Hub area quantification was determined by drawing a line around the *z*-slice with the largest hub size based on Armadillo or Fasciclin III immunostaining and using the area measurement in Fiji. For the early germ cell proliferation phenotype, Armadillo was used as the marker to delineate the germline cysts (Feng et al., 2017). Any cyst with more than 16 cells was considered overpopulated.

### Statistics and reproducibility

Data were subjected to the Shapiro–Wilk test to determine whether the data were normally distributed or skewed. For normally distributed data, an unpaired, two-sample *t*-test was used to compare two individual datasets with each other. For skewed data, the Wilcoxon signed rank test was used to compare two individual datasets with each other. A χ^2^ test was used to compare distributions of datasets containing data from different categories. Two-way ANOVA with interaction and Šidák multiple comparison test were used to compare two individual datasets with each other that had two independent variables. One-way ANOVA and Dunnett's T3 multiple comparison test were used when more than two individual datasets were compared with each other that had one independent variable. Data are presented with error bars representing the s.e.m. Significant differences based on these statistical analyses are noted by asterisks (*P<0.05, **P<10^−2^, ***P<10^−3^, ****P<10^−4^).

## Supplementary Material



10.1242/develop.202729_sup1 Supplementary information

Table S1. Quantification of Germline Stem Cell number in RNAi knockdown testes. nanos-Gal4 driven Control RNAi data
nanos-Gal4 driven set1 RNAi data
nos-Gal4, tub-Gal80^ts^ driven Control RNAi data
nos-Gal4, tub-Gal80^ts^ driven set1 RNAi data
tj-Gal4 driven RNAi data
bam-Gal4 driven RNAi data
nos-Gal4, bam-Gal80 driven RNAi data
Genetic interaction data

Table S2. Quantification of the hub area in RNAi knockdown testes. nanos-Gal4 driven Control RNAi data
nanos-Gal4 driven set1 RNAi data
nos-Gal4, tub-Gal80^ts^ driven Control RNAi data
nos-Gal4, tub-Gal80^ts^ driven set1 RNAi data
tj-Gal4 driven RNAi data
bam-Gal4 driven RNAi data
nos-Gal4, bam-Gal80 driven RNAi data

Table S3. Quantification of Cyst Cell number in RNAi knockdown testes. nanos-Gal4 driven Control RNAi data
nanos-Gal4 driven set1 RNAi data
nos-Gal4, tub-Gal80^ts^ driven Control RNAi data
nos-Gal4, tub-Gal80^ts^ driven set1 RNAi data
tj-Gal4 driven RNAi data
bam-Gal4 driven RNAi data
nos-Gal4, bam-Gal80 driven RNAi data
