# Supplementary material for: The Drosophila histone methyltransferase SET1 coordinates multiple signaling pathways in regulating male germline stem cell maintenance and differentiation
Source: Development. 2024 Aug 9;151(15):dev202729. doi: 10.1242/dev.202729 (PMC11369688; doi:10.1242/dev.202729)
Supplement: Supplementary information [file develop-151-202729-s1.pdf]

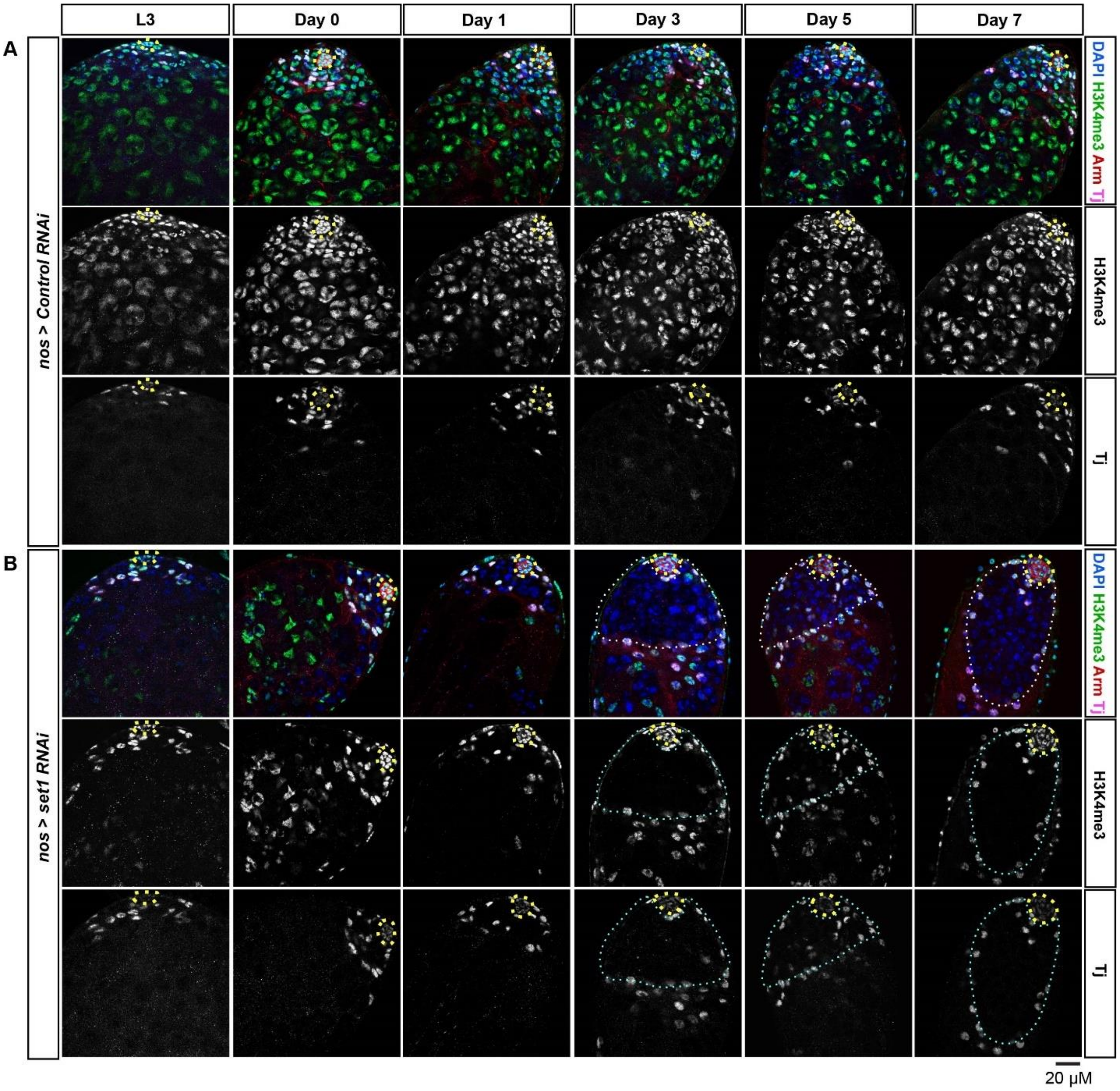

Fig. S1. H3K4me3 in the *Drosophila* testis is dependent on Set1. Representative images of *nos>Control RNAi* testes (**A**) and *nos>set1 RNAi* testes (**B**) at L3, day 0, 1, 3, 5 and 7 post eclosion immunostained with H3K4me3 (green), Arm (red) for the hub region (yellow dotted outline), and Tj (magenta) for the CySC lineage cells, DAPI (blue). Cyan dotted outlines: overpopulated early-stage germ cells. Scale: 20 μm.

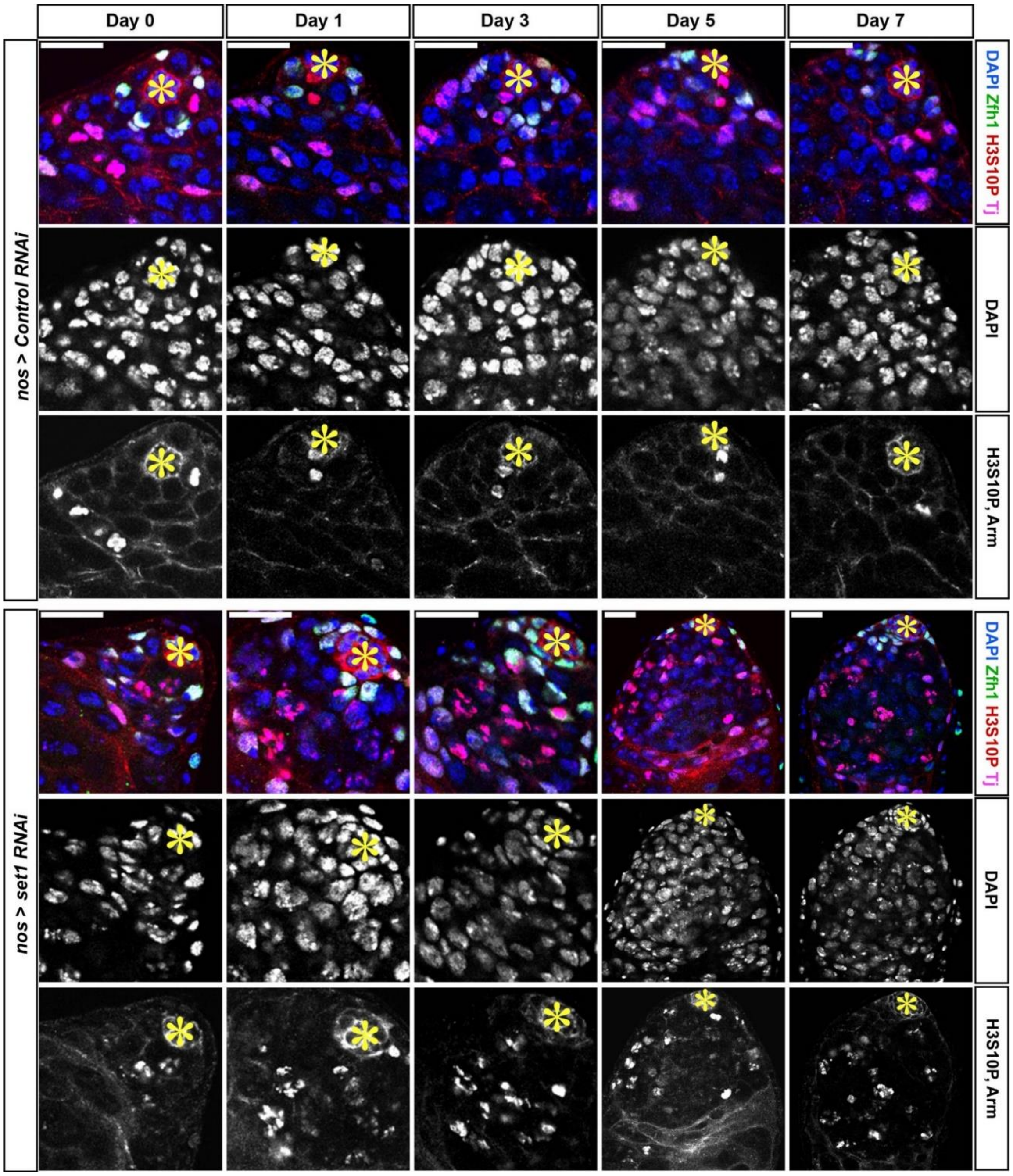

Fig. S2. Increased H3S10ph-positive germ cells in the *set1* knockdown *Drosophila* testis.

Representative images of *nos>Control RNAi* testes (**A**) and *nos>set1 RNAi* testes (**B**) at day 0, 1, 3, 5 and 7 post eclosion immunostained with Zfh1 (green), Arm (red) for the hub region (yellow Asterisk), H3S10P (red) for mitotic cells, Tj (magenta) for the CySC lineage cells, and DAPI (blue). Scale: 20 μm.

Development • Supplementary information

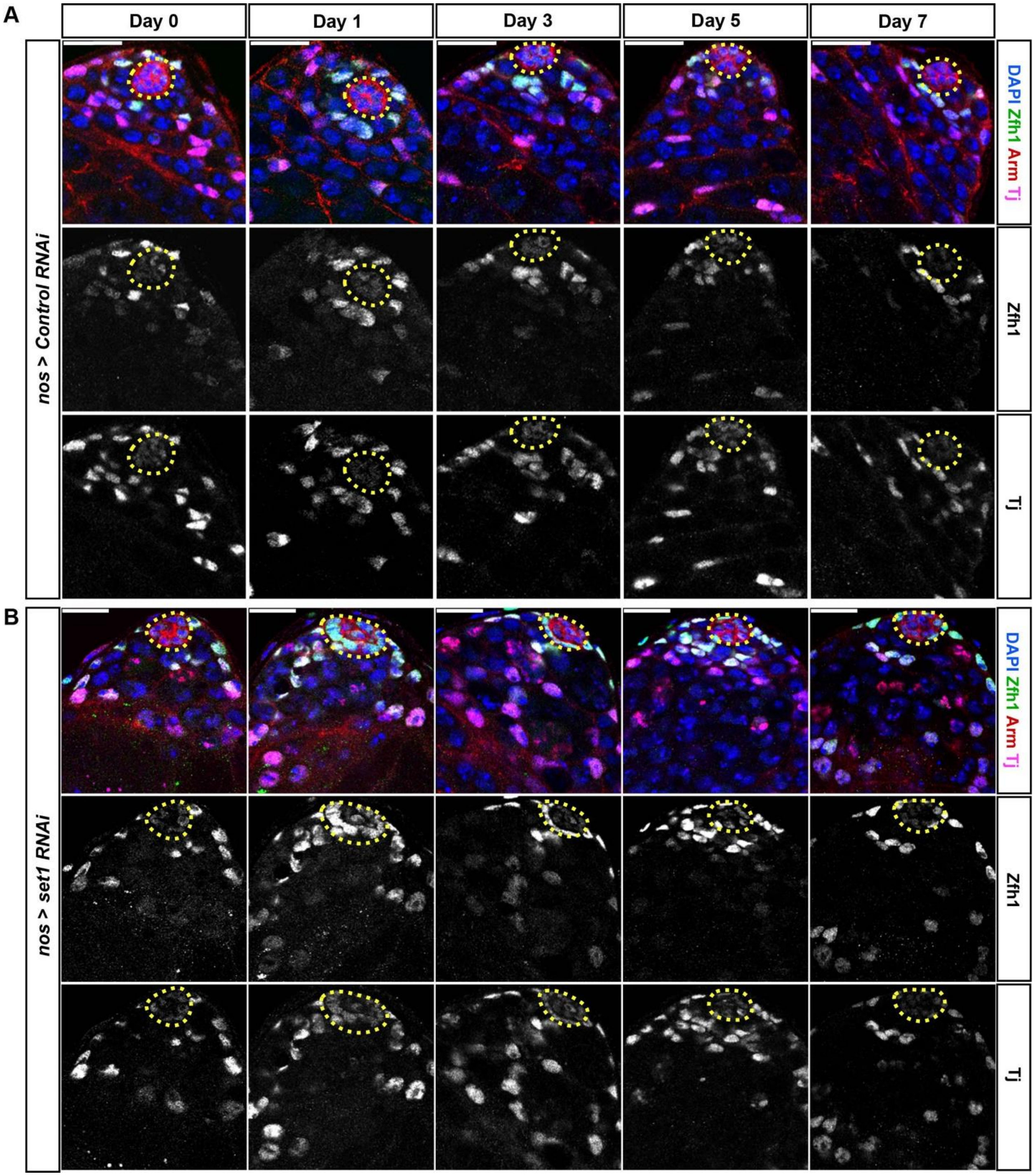

Fig. S3. Zfh1 expression in *Control* RNAi and *set1* RNAi *Drosophila* testes.

Representative images of *nos>Control RNAi* testes (**A**) and *nos>set1 RNAi* testes (**B**) at day 0, 1, 3, 5 and 7 post eclosion immunostained with Zfh1 (green), Arm (red) for the hub region (yellow dotted outline), and Tj (magenta) for the CySC lineage cells, DAPI (blue). Scale: 20 μm.

Development • Supplementary information

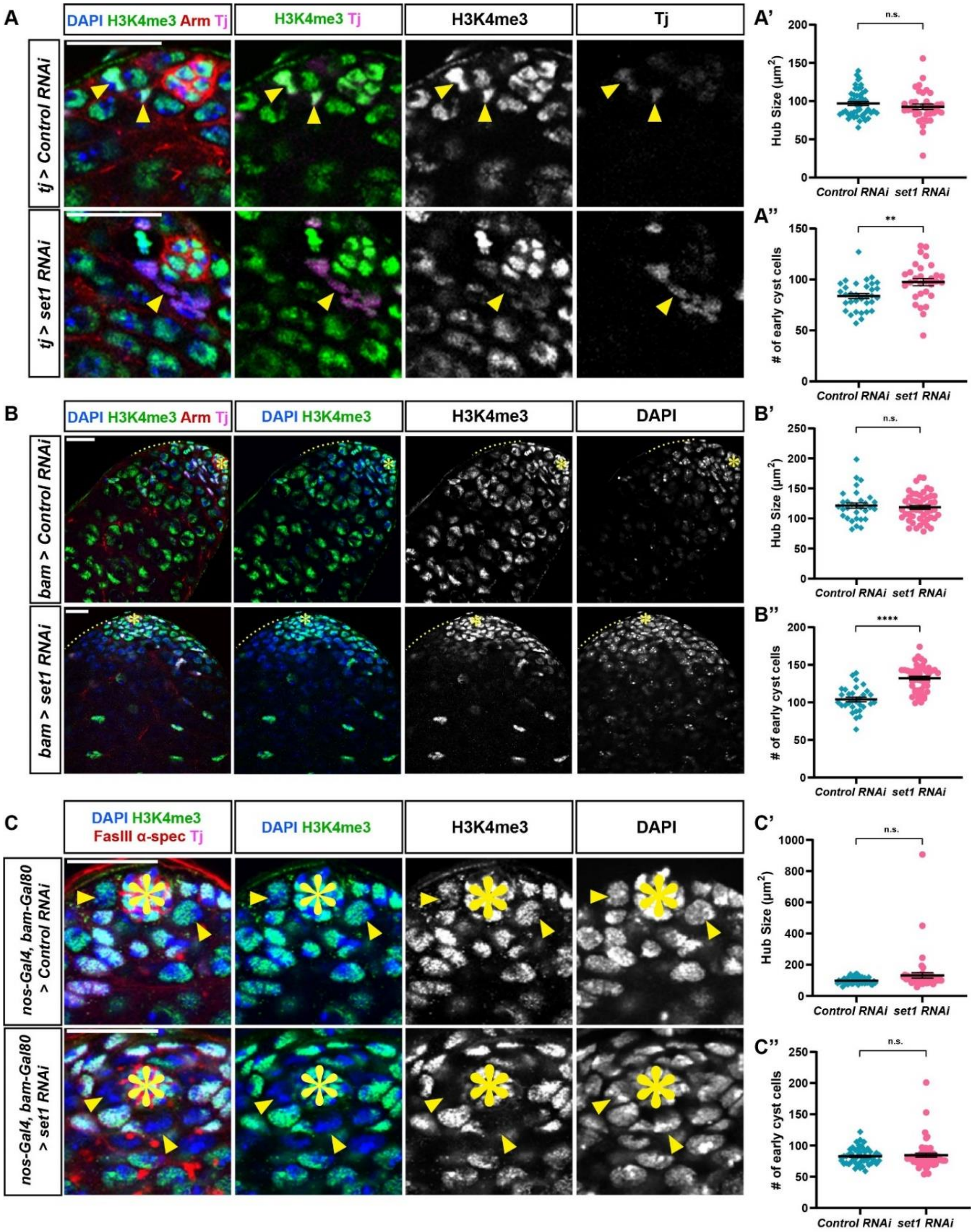
A
DAPI H3K4me3 Arm Tj
H3K4me3 Tj
H3K4me3
Tj
tj > Control RNAi
tj > set1 RNAi
A'
n.s.
Hub Size (μm2)
Control RNAi
set1 RNAi
A''
**
# of early cyst cells
B
DAPI H3K4me3 Arm Tj
DAPI H3K4me3
H3K4me3
DAPI
bam > Control RNAi
bam > set1 RNAi
B'
B''
****
C
DAPI H3K4me3
FasIII α-spec Tj
nos-Gal4, bam-Gal80 > Control RNAi
nos-Gal4, bam-Gal80 > set1 RNAi
C'
C''

Fig. S4. H3K4me3 signals are reduced in the somatic gonadal cells, late spermatogonial cells, and GSCs in the *Drosophila* testis by knockdown of *set1* using cell type- and stage-specific drivers. (**A**) Representative images of *tj>Control RNAi* and *tj>set1 RNAi* testes at day 7 post eclosion, immunostained with H3K4me3 (green), Arm (red) for the hub region, and Tj (magenta) for the CySC lineage cells. (**A'**) Quantification of the hub region size for *tj>Control RNAi* testes (N= 46) and *tj>set1 RNAi* testes (N= 36). Refer to Table S2. (**A''**) Quantification of cyst cell number for *tj>Control RNAi* (N= 33) and *tj>set1 RNAi* (N= 30) testes. Individual data points and mean values are shown. Refer to Table S3. (**B**) Representative images of *bam>Control RNAi* and *bam>set1 RNAi* testes at day 7 post eclosion, immunostained with H3K4me3 (green), Arm (red) for the hub region, and Tj (magenta) for the CySC lineage cells. (**B'**) Quantification of the hub size for *bam>Control RNAi* testes (N= 31) and *bam>set1 RNAi* testes (N= 50). Refer to Table S2. (**B''**) Quantification of cyst cell number for *bam>Control RNAi* and *bam>set1 RNAi* testes. Refer to Table S3. (**C**) Representative images of *nos-Gal4ΔVP16, bam-Gal80 >Control RNAi* and *nos-Gal4ΔVP16, bam-Gal80 >set1 RNAi* testes at day 7 post eclosion, immunostained with H3K4me3 (green), Fas III (red) for the hub region, α-Spectrin (red) for spectrosome and fusome, and Tj (magenta) for the CySC lineage cells. (**C'**) Quantification of hub region size for *nos-Gal4ΔVP16, bam-Gal80 >Control RNAi* testes (N= 52) and *nos-Gal4ΔVP16, bam-Gal80 >set1 RNAi* testes (N= 52). Refer to Table S2. (**C''**) Quantification of cyst cell number for *nos-Gal4ΔVP16, bam-Gal80>Control RNAi* and *nos-Gal4ΔVP16, bam-Gal80>set1 RNAi* testes. Refer to Table S3. (**A'-A", B'-B", C'-C"**) Individual data points and mean values are shown. Error bars represent SEM. ****$P < 10^{-4}$, **$P < 10^{-2}$, n.s.: not significant, unpaired t test to compare two individual datasets to each other. Asterisk in (**B** and **C**): Hub region. Yellow arrow heads: cyst cells in (**A**); GSCs in (**C**). Yellow dotted line: 4-16 spermatogonial cell region in (**B**). Scale: 20 μm.

Development • Supplementary information

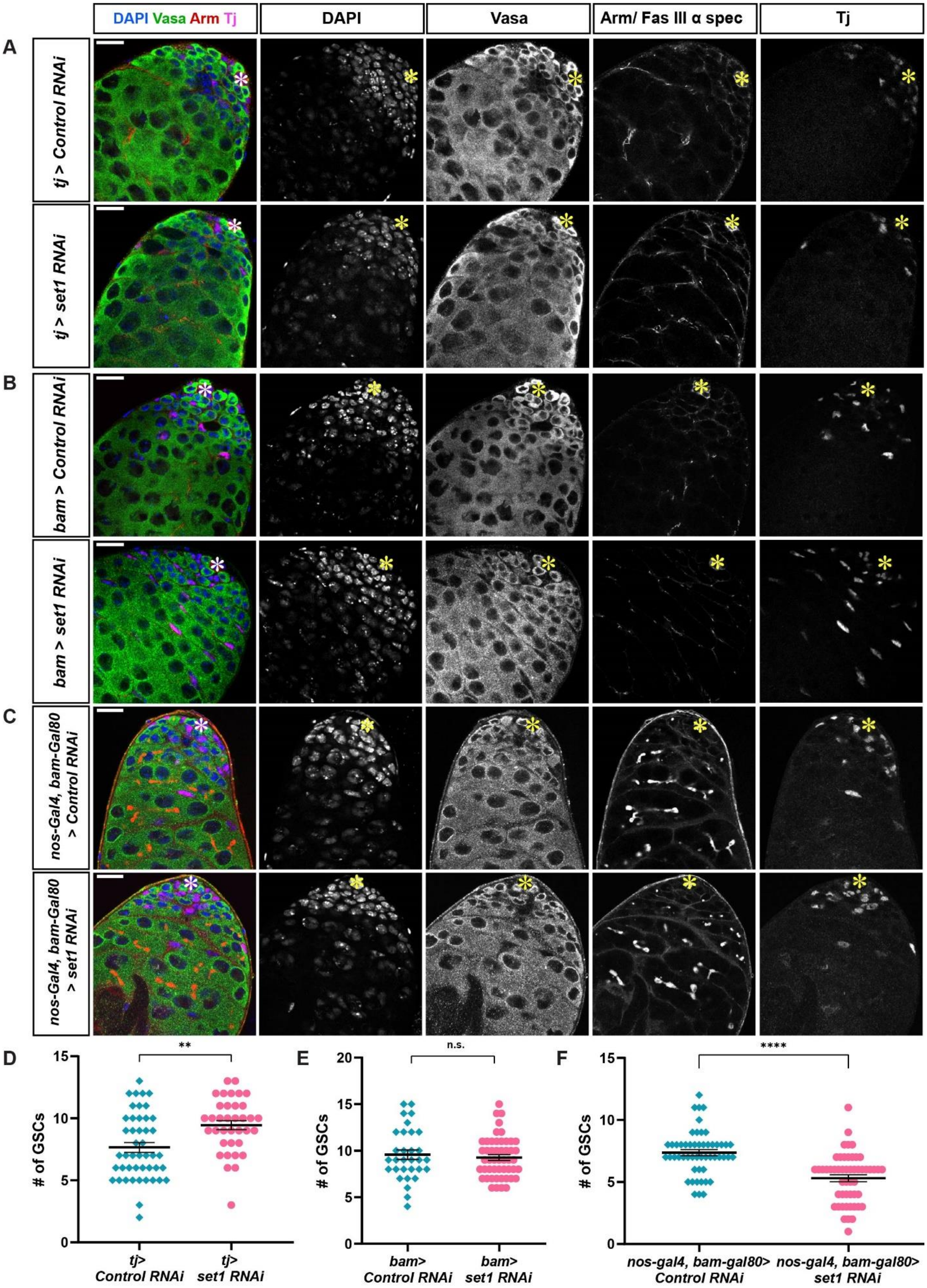

Development • Supplementary information

Fig. S5. Set1 is dispensable in somatic gonadal and spermatogonial cells to regulate GSC maintenance. (**A**) Representative images of *tj>Control RNAi* and *tj>set1 RNAi* testes at 7 days post eclosion. (**B**) Representative images of *bam>Control RNAi* and *bam>set1 RNAi* testes at 7 days post eclosion. (**C**) Representative images of *nos-Gal4ΔVP16, bam-Gal80>Control RNAi* and *nos-Gal4ΔVP16, bam-Gal80>set1 RNAi* testes at 7 days post eclosion. All images in (**A-C**) stained with germ cell marker Vasa (green), Arm (red) or Fas III for the hub area (yellow asterisk), α-Spectrin (red) for spectrosome and fusome, and Tj (magenta) for the CySC lineage cells. Scale: 20 μm. (**D**) Quantification of GSC number for *tj>Control RNAi* testes (N = 46) and *tj>set1 RNAi* testes (N = 36). (**E**) Quantification of GSC number for *bam>Control RNAi* testes (N = 31) and *bam>set1 RNAi* testes (N = 50). (**F**) Quantification of GSC number for *nos-Gal4ΔVP16, bam-Gal80>Control RNAi* testes (N = 52) and *nos-Gal4ΔVP16, bam-Gal80>set1 RNAi* testes (N = 52). For (**D-F**): Individual data points and mean values are shown. Refer to Table S1. Error bars represent SEM. ****$P<10^{-4}$, ** $P<10^{-2}$, n.s.: not significant; unpaired t test to compare two individual datasets to each other.

Development • Supplementary information

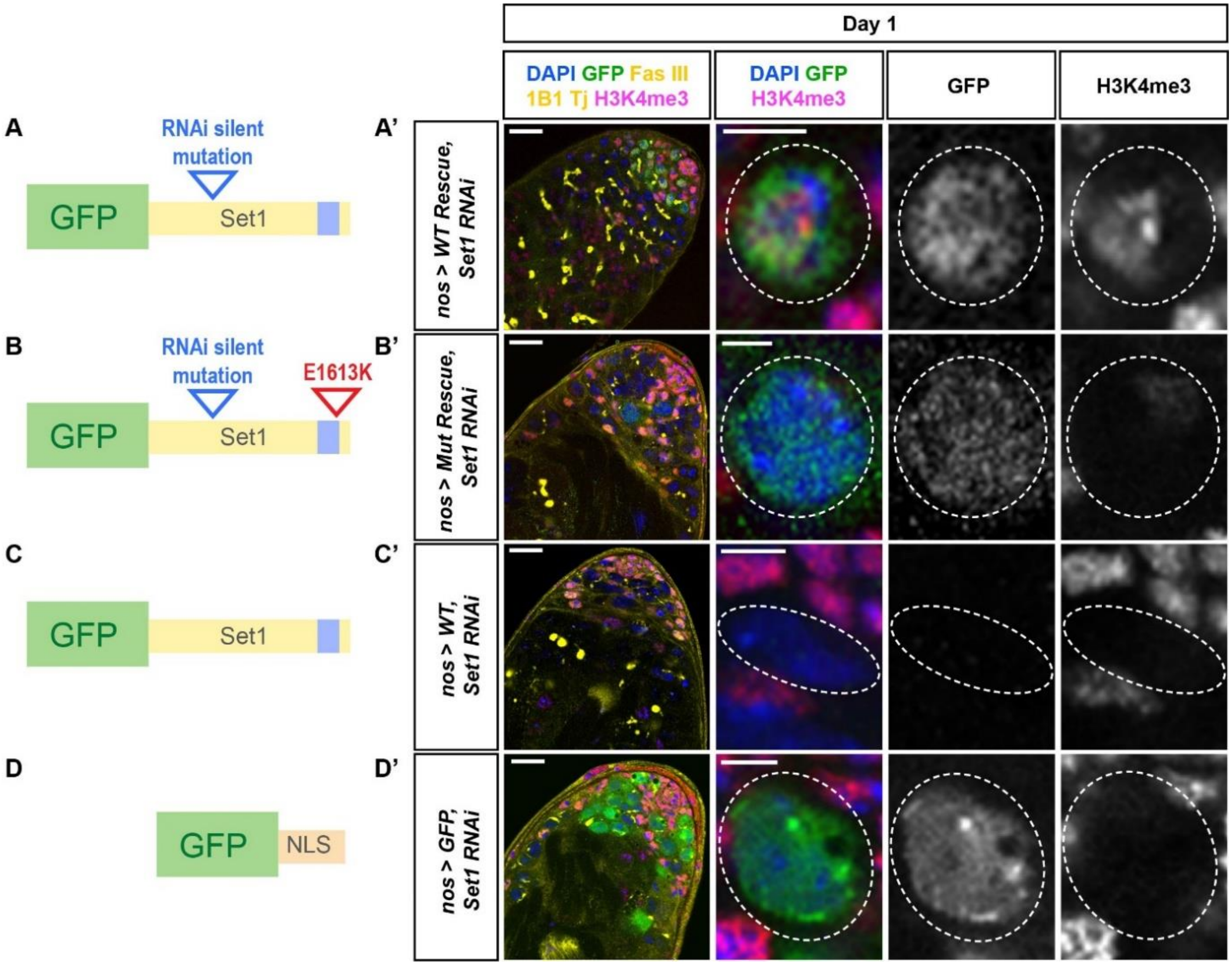

Fig. S6. The methyltransferase activity of Set1 is required for its function in the male germline. (**A**-**D**) Cartoon depictions: the *set1* cDNA transgene with the RNAi recognition sequences mutated, named WT rescue (**A**); the *set1* cDNA transgene with the RNAi recognition sequences mutated and an E→K amino acid change in the SET domain, named Mut rescue (**B**); the *set1* cDNA transgene without the RNAi recognition sequences mutated, named WT (**C**); the *GFP* cDNA with the *nuclear localization* sequence, named GFP (**D**). Representative images of *nos>WT Rescue*, *set1 RNAi* testis (**A'**), *nos>Mut Rescue*, *set1 RNAi* testis (**B'**), *nos>WT*, *set1 RNAi* testis (**C'**), *nos>GFP*, *set1 RNAi* testis (**D'**), all testes are from males 1 day post eclosion,

Development • Supplementary information

immunostained with H3K4me3 (magenta), GFP (green), Fas III for the hub region, 1B1 (yellow) for spectrosome and fusome, and Tj (yellow) for the CySC lineage cells. White dotted outline: germ cell expressing the corresponding transgenes. Scale: 20 μm for the testis image; 5 μm for individual germ cell images.

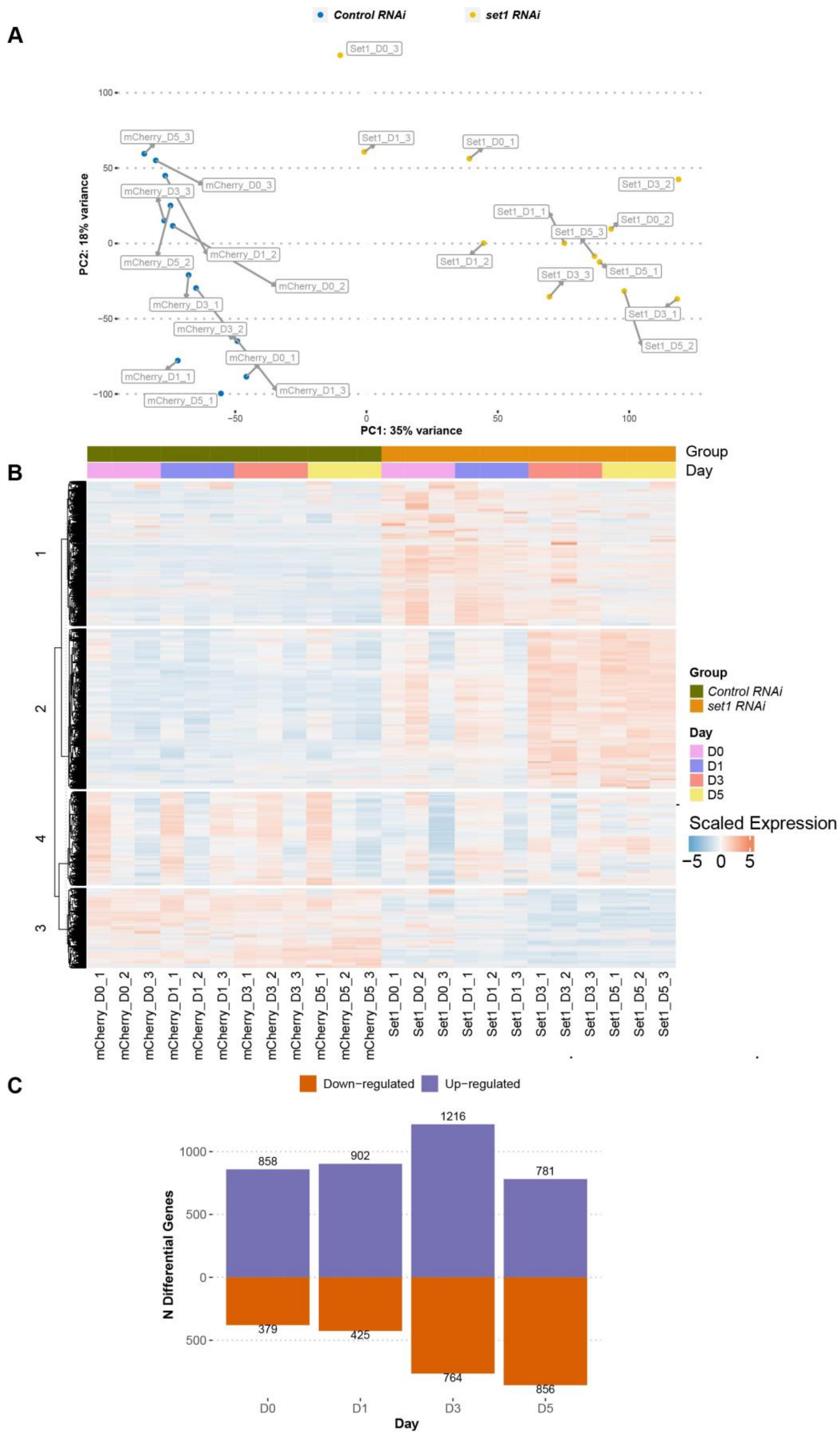
A
Control RNAi
set1 RNAi
PC1: 35% variance
PC2: 18% variance
B
Group
Day
Control RNAi
set1 RNAi
D0
D1
D3
D5
Scaled Expression
−5 0 5
C
Down−regulated
Up−regulated
N Differential Genes
Day
858
902
1216
781
379
425
764
856

D

Gene	Log2 FC	Adjusted P-value
JAK-STAT signaling pathway		
upd1	-0.85	2.82E-04
upd2	1.26	0.06
hop	0.52	0.03
dome	0.16	0.49
BMP signaling pathway		
gbb	0.70	4.45E-03
mad	0.03	0.89
med	0.80	6.14E-05
put	1.26	4.86E-09
sax	-0.29	0.20
tkv	0.62	4.89E-03
EGF signaling pathway		
vn	0.98	2.37E-03
grk	0.67	0.03
krn	0.02	0.93
spi	0.40	0.04
Dsor1	1.28	2.32E-08
egfr	1.35	1.07E-06
raf	0.72	5.24E-03
rl	1.08	3.21E-05
pnt	1.50	1.16E-09
Ras85D	1.01	3.53E-06
drk	0.37	0.05

Gene	Log2 FC	Adjusted P-value
Notch signaling pathway		
N	0.53	0.03
Su(H)	-0.83	2.29E-04
Ser	-1.22	3.07E-08
mam	0.94	6.87E-05
Hedgehog signaling pathway		
hh	-0.03	0.93
ptc	0.19	0.54
smo	1.19	2.04E-05
ci	0.67	0.02
ihog	0.75	0.06
fu	0.96	4.21E-03
cos	0.64	0.01
Wnt signaling pathway		
wg	0.55	0.29
arm	0.64	7.52E-04
arr	0.14	0.60
dsh	-0.34	0.06
fz	1.34	1.19E-05
fz2	1.20	3.14E-08
Hippo signaling pathway		
hpo	0.68	1.01E-03
mats	-0.37	0.04
sav	-0.13	0.57
wts	0.38	0.09
yki	-0.57	5.48E-03

Fig. S7. Knockdown of *set1* in the early germline results in global gene expression changes. (**A**) Principal component analysis plot shows multidimensional distribution of all *nos>set1 RNAi* (*set1* KD, 12 samples) and *nos>Control RNAi* (*Ctrl* KD, 12 samples) data sets: Three biological replicates for both *set1* KD and *Ctrl* KD at day 0, 1, 3, 5 post eclosion,

Development • Supplementary information

respectively. With three biological replicates, two genotypes and four time points, we analyzed all 24 samples. There are two main clusters: a *set1* KD cluster and a *Ctrl* KD cluster. Within the *set1* KD cluster, Day 0 and Day 1 timepoint samples cluster while Day 3 and Day 5 timepoint samples cluster. (**B**) Heatmap shows differentially expressed genes (DEGs) between *set1* KD and *Ctrl* KD testes at day 0, 1, 3, 5 post eclosion, respectively. All genes are separated into four different groups. Group 1 contains the genes that are most expressed in *set1* KD testes at Day 0 and 1. Group 2 consists of the genes that are most expressed in *set1* KD testes at Day 3 and 5. Group 3 is comprised of genes more highly expressed in *Ctrl* KD testes and Group 4 is composed of the genes that did not fit any particular expression pattern with regards to genotype or timepoint. (**C**) Bar plot shows the number of DEGs with ≥1.3-fold (log_2 scale) and $P< 0.05$ in *set1* KD testes compared to *Ctrl* KD testes at day 0, 1, 3, 5 post eclosion, respectively. (**D**) A table shows the detailed expression changes of several core components of the JAK-STAT, BMP, EGF, Notch, Hedgehog, Wnt, and Hippo signaling pathways at Day 3. Genes that are significantly upregulated are highlighted in purple and genes that were significantly downregulated are highlighted in orange.

Development • Supplementary information

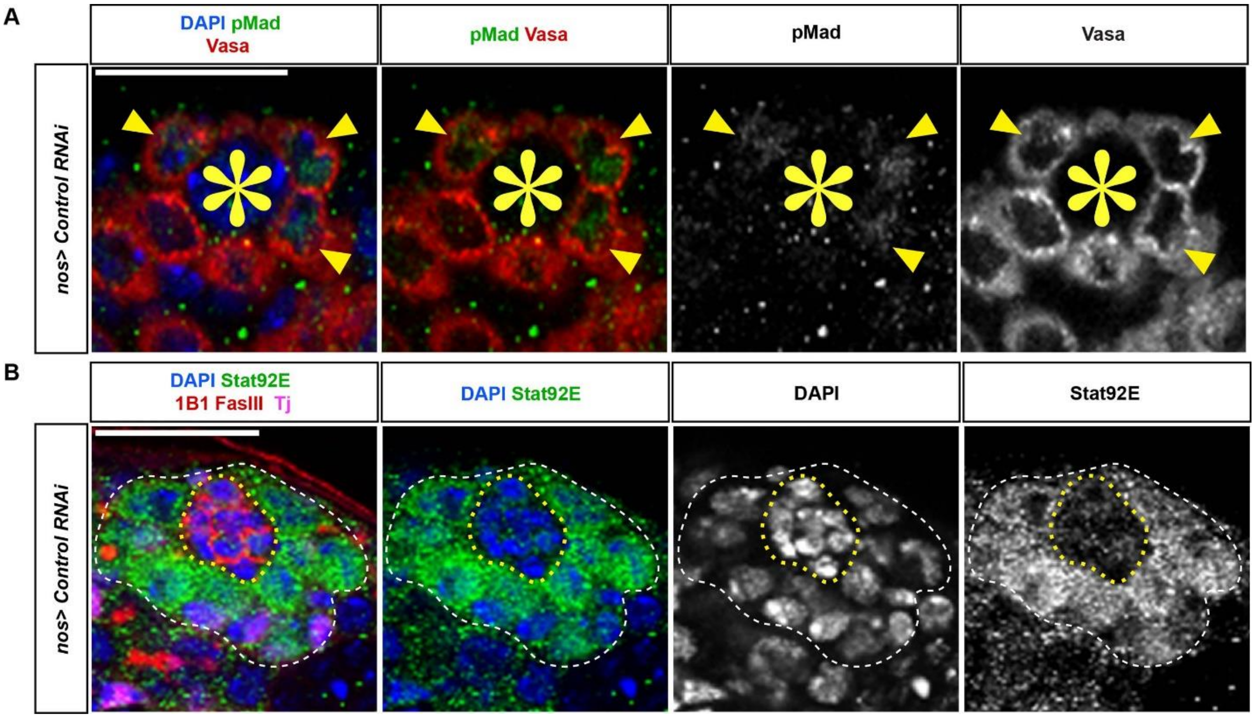

Fig. S8. The immunostaining results of pMad and Stat92E in *Control* RNAi *Drosophila* testes. (**A**) Representative images of *nos-Gal4>Control RNAi* testes immunostained with pMad (green), Vasa (red) for the germ cells, and DAPI (blue). In (**A**): yellow asterisk: hub; yellow arrows: pMad positive GSCs. (**B**) Representative images of *nos-Gal4>Control RNAi* testes immunostained with Stat92E (green), Fas III (red) for the hub region, 1B1 (red) for spectrosome and fusome, and Tj (magenta) for the CySC lineage cells, and DAPI (blue). In (**B**): yellow dotted outline: hub; white dotted outline: the GSCs and CySCs region. Scale: 20 μm.

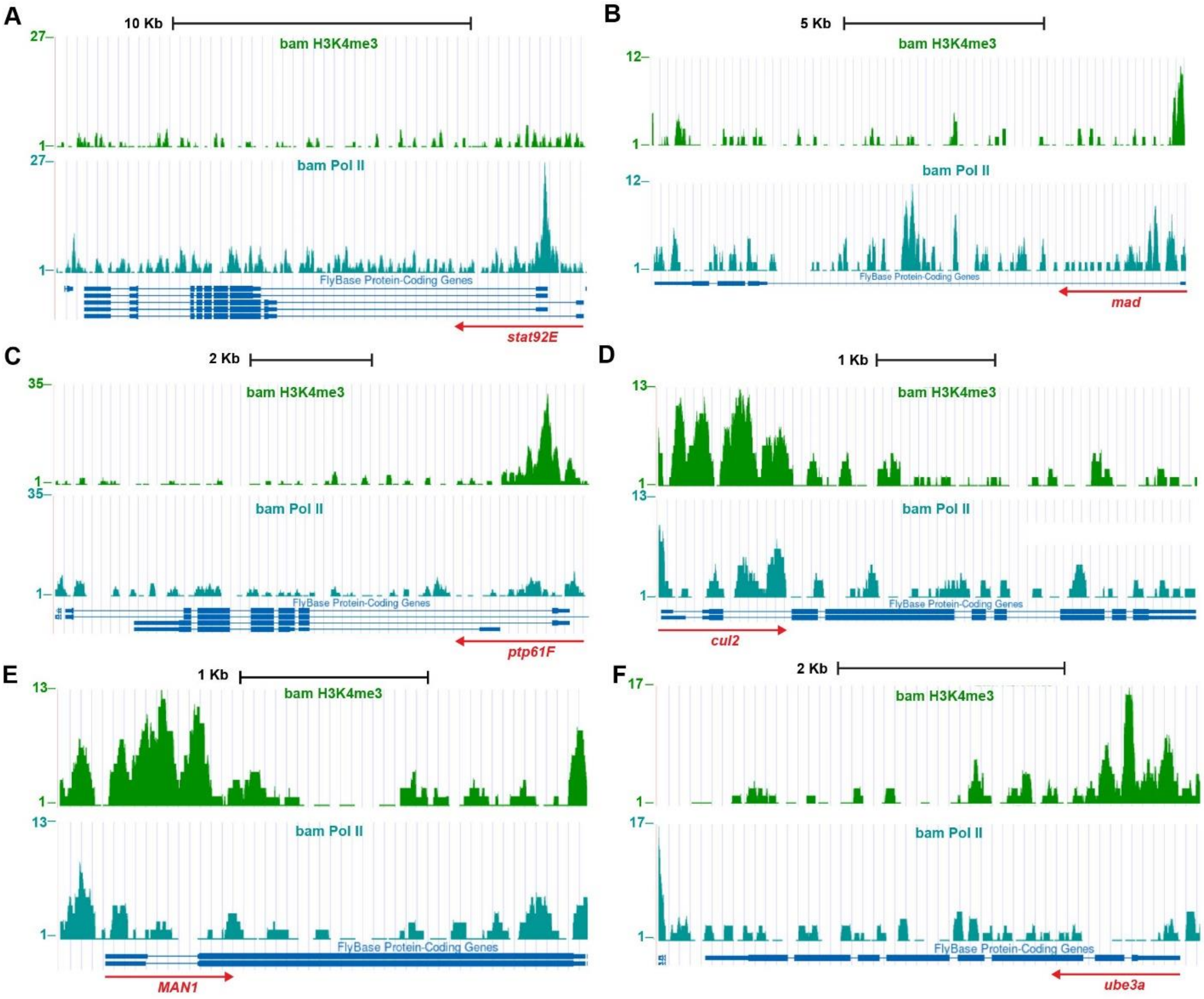

G

Gene	Log2 FC	Adjusted P-value
Negative regulators of JAK-STAT signaling pathway		
ptp61F	-0.14	0.51
Negative regulators of BMP signaling pathway		
cul2	-0.15	0.43
MAN1	-0.35	0.08
ube3a	-0.04	0.86

Development • Supplementary information

Fig. S9. UCSC genome browser screenshots show H3K4me3 and RNA Pol II enrichment at individual gene regions. (**A-F**) All data are based on ChIP-seq using progenitor germ cell-enriched *bam* mutant testes (Gan et al., 2010), H3K4me3 (green) and RNA polymerase II (blue). (**A**) At the *stat92E* gene locus, H3K4me3 is not distinctly enriched at the promoter region even though Pol II profiles indicates active transcription. (**B**) At the *mad* gene locus, H3K4me3 is enriched at the promoter region and Pol II profiles indicates active transcription. (**C**) At the *ptp61F* gene locus, H3K4me3 is very distinctly enriched at the promoter region, even though Pol II profiles indicates inactive transcription. (**D**) At the *cul-2* gene locus, H3K4me3 is enriched at the promoter region and Pol II profiles indicates active transcription. (**E**) At the *MAN1* gene locus, H3K4me3 is enriched at the promoter region and Pol II profiles indicates active transcription. (**F**) At the *ube3a* gene locus, H3K4me3 is very distinctly enriched at the promoter region, even though Pol II profiles indicates inactive transcription. (**G**) A table shows the detailed expression changes of several inhibitors of the JAK-STAT and BMP signaling pathways at Day 3.

Development • Supplementary information

Table S1. Quantification of Germline Stem Cell number in RNAi knockdown testes.

nanos-Gal4 driven Control RNAi data

nanos-Gal4 driven set1 RNAi data

nos-Gal4, tub-Gal80^{ts} driven Control RNAi data

nos-Gal4, tub-Gal80^{ts} driven set1 RNAi data

tj-Gal4 driven RNAi data

bam-Gal4 driven RNAi data

nos-Gal4, bam-Gal80 driven RNAi data

Genetic interaction data

Available for download at
https://journals.biologists.com/dev/article-lookup/doi/10.1242/dev.202729#supplementary-data

Table S2. Quantification of the hub area in RNAi knockdown testes.

nanos-Gal4 driven Control RNAi data

nanos-Gal4 driven set1 RNAi data

nos-Gal4, tub-Gal80^{ts} driven Control RNAi data

nos-Gal4, tub-Gal80^{ts} driven set1 RNAi data

tj-Gal4 driven RNAi data

bam-Gal4 driven RNAi data

nos-Gal4, bam-Gal80 driven RNAi data

Available for download at
https://journals.biologists.com/dev/article-lookup/doi/10.1242/dev.202729#supplementary-data

Table S3. Quantification of Cyst Cell number in RNAi knockdown testes.

nanos-Gal4 driven Control RNAi data

nanos-Gal4 driven set1 RNAi data

nos-Gal4, tub-Gal80^{ts} driven Control RNAi data

nos-Gal4, tub-Gal80^{ts} driven set1 RNAi data

tj-Gal4 driven RNAi data

bam-Gal4 driven RNAi data

nos-Gal4, bam-Gal80 driven RNAi data

Available for download at
https://journals.biologists.com/dev/article-lookup/doi/10.1242/dev.202729#supplementary-data

Supplemental References

Gan, Q., Schones, D.E., Ho Eun, S., Wei, G., Cui, K., Zhao, K., Chen, X., 2010. Monovalent and unpoised status of most genes in undifferentiated cell-enriched Drosophila testis. Genome Biol 11, R42.
