# Supplementary material for: The Drosophila histone methyltransferase SET1 coordinates multiple signaling pathways in regulating male germline stem cell maintenance and differentiation
Source: Development. 2024 Aug 9;151(15):dev202729. doi: 10.1242/dev.202729 (PMC11369688; doi:10.1242/dev.202729)
Supplement: Supplementary file 1 [file develop-151-202729_review_history.pdf]

The *Drosophila* histone methyl-transferase SET1 coordinates multiple signaling pathways in regulating male germline stem cell maintenance and differentiation

Velinda Vidaurre, Annabelle Song, Taibo Li, Wai Lim Ku, Keji Zhao, Jiang Qian and Xin Chen

DOI: 10.1242/dev.202729

Editor: Haruhiko Koseki

Review timeline

Original submission:	25 January 2024
Editorial decision:	18 March 2024
First revision received:	25 June 2024
Accepted:	8 July 2024

Original submission

First decision letter

MS ID#: DEVELOP/2024/202729

MS TITLE: The Drosophila histone methyl-transferase SET1 coordinates multiple signaling pathways in regulating male germline stem cell maintenance and differentiation

AUTHORS: Velinda Vidaurre, Annabelle Song, Taibo Li, Wai Lim Ku, Keji Zhao, Jiang Qian, and Xin Chen

I have now received all the referees' reports on the above manuscript, and have reached a decision. The referees' comments are appended below, or you can access them online: please go to BenchPress and click on the 'Manuscripts with Decisions' queue in the Author Area.

As you will see, the referees express considerable interest in your work, but have some significant criticisms and recommend a substantial revision of your manuscript before we can consider publication. If you are able to revise the manuscript along the lines suggested, which may involve further experiments, I will be happy receive a revised version of the manuscript. Your revised paper will be re-reviewed by one or more of the original referees, and acceptance of your manuscript will depend on your addressing satisfactorily the reviewers' major concerns. Please also note that Development will normally permit only one round of major revision. If it would be helpful, you are welcome to contact us to discuss your revision in greater detail. Please send us a point-by-point response indicating your plans for addressing the referees’ comments, and we will look over this and provide further guidance.

Please attend to all of the reviewers' comments and ensure that you clearly highlight all changes made in the revised manuscript. Please avoid using 'Tracked changes' in Word files as these are lost in PDF conversion. I should be grateful if you would also provide a point-by-point response detailing how you have dealt with the points raised by the reviewers in the 'Response to Reviewers' box. If you do not agree with any of their criticisms or suggestions please explain clearly why this is so.

Reviewer 1

© 2024. Published by The Company of Biologists under the terms of the Creative Commons Attribution License (https://creativecommons.org/licenses/by/4.0/).

Advance summary and potential significance to field

Proper regulation of stem cell lineages is important for tissue function. Stem cell maintenance and differentiation can be regulated by external as well as internal factors, such as epigenetic regulators. In this manuscript, Vidaurre et al propose that the histone methyltransferase Set1 regulates germline stem cell (GSC) maintenance and progeny differentiation in the adult Drosophila testis. The authors provide evidence that knockdown of Set1 specifically in the germline decreases GSC numbers and that early differentiating cysts overpopulate and display tumor phenotypes. The authors further propose that loss of set1 enhances JAK/STAT and BMP signaling. The findings from this study will be of interest to researchers in the epigenetic, stem cell, and cancer-related fields.

Comments for the author

I found this manuscript and the findings from the authors extremely interesting; however I do have suggestions and comments regarding statistical analyses, quantification, and proper controls used for the experiments performed in the current version of the manuscript.

Major points:
1. The statistical analysis using an unpaired t-test does not seem appropriate. Because the different genotypes could have different starting points (e.g., different GSC numbers to begin with), it is unfair to do a direct comparison between the control and set1 RNAi. For the time course experiments, it would be better to perform an ANOVA with interaction. Furthermore, the one timepoint only analyses have the same problem (we don't know where the GSC/cyst cell numbers/etc started out at day 0, so statements suggesting that GSC numbers are higher or lower aren't fully accurate.
2. Please define the parameters for overpopulation (never defined in the text). How is this determined? Is it based on the number of 2-, 4-, 8-cell cysts per GSC? The authors would need to determine this to say whether or not there are more early stage cysts in the set1 RNAi conditions compared to control.
3. How can the authors determine if the phenotypes are due to over proliferation (e.g., red label in graph for Figure 1C) and not de-differentiation of cysts? Labeling with EdU or pHH3 would need to be performed to say it is over proliferation.
4. The authors suggest that GSC maintenance is affected with set1 is knocked down in the germline during development (Figure 1E). However, if you look at the slopes, the maintenance of the GSCs is not different compared to the control – the different genotypes simply start out at different points. The more convincing data regarding maintenance is found in the supplemental with the adult-specific manipulations. I would recommend either moving the adult analysis to the main figures or re-interpreting the developmental knockdown conclusions.
5. The phenotypes observed with set1 RNAi seem to be more exaggerated when manipulation occurs during development. What do the larval and pupal testis look like compared to control?
6. How do the authors distinguish early cysts from late cysts? The methods say that the early cyst number was quantified by counting every Tj positive cell. Wouldn't that include late cysts?
7. The authors mention in the text (page 10) that differences observed in the phenotypes could be due to differences in knockdown strength. It is unclear why additional RNAi lines were not used (two TRiP lines available in the BDSC, and two different lines available in the VDRC).
8. How do the authors quantify/determine "normal morphology" (Figure 5C, page 14)? Some of the nos>set1 RNAi images look similarly "normal."
9. For the genetic interaction analysis using the stat92E[06346] and mad[12] alleles, a better control would be to have these alleles in the background of the nos-Gal4 driver (Figure 5).
10. Fluorescence intensities in Figures 5A and 5B should be quantified for pMad and Stat92E levels.
11. The argument that set1 RNAi influences negative regulators of JAK/STAT and BMP signaling would be strengthened if rescue experiments were performed. There are UAS constructs available in the BDSC for Ptp61F and Ube3.

Minor points:
1. Why do the authors only perform two biological replicates instead of at least three?
2. The Vasa staining for the 21 and 28 day set1 RNAi images is missing.

© 2024. Published by The Company of Biologists under the terms of the Creative Commons Attribution License (https://creativecommons.org/licenses/by/4.0/).

Reviewer 2

Advance summary and potential significance to field

In this manuscript by Velinda Vidaurre et al., the authors make an interesting observation regarding different functions of the same gene product, Set1, in different steps of the differentiation of Drosophila germline stem cells.
They show that knockdown of Set1 induced in adult germ cells leads first, to the loss of more differentiated germ cells, followed by over-proliferation of the early germ cells themselves. They implicate the Jak-stat and BMP signalling pathway data based on RNA seq data.

Comments for the author

While the manuscript is convincing at the face of it, I recommend the following additions and amendments for rigour and alleviating doubt:

1. Use of a single RNAI line: In the study, the loss of Set1 is achieved by the use of a single RNAi line.
Formalism requires at least three indelpendent lines with at least one rescue, both of the phenotype and the posited disrupted mechanism. Experiments shown in figure 3 with the catalytically inactive Set1, do suggest that phenotype may be due to Set1b loss of function alone. However, because subsequent analysis is based on bulk RNA-seq of the knockdown tissue, where off target effects will undoubtedly manifest, I suggest the phenotype be reproduced with at least one more RNAi Line and the RNA seq data show agreement with some identified targets. This experiment may be essential in view of transcriptional compensation that has been previously reported.
2. JNK and BMP as effectors of Set1 KD phenotype: As the authors note JNK and BMP are known pathways regulating stem cell maintenance. The RNAi KD show great statistically significant differences in their levels upon downregulation. However, the fold changes are between 2 and 4 times. Their assertion is supported by the modification of the set1 KD phenotype being modified by stat92E alleles. The modification, though, is cell clearance, which may be cell death. Many perturbations can enhance the clearance of cells, so the assay for epistasis analysis may not be well-founded.
In this regard, I have two suggestions: i) RNAseq of KD tissue from a second independent RNAi line to check if the same targets filter through (same experiment as the previous point). ii) At least an analysis of other candidate regulatory pathways so that the field may go beyond the known pathways that have been dwelled on for decades. No experimental verification is needed for this manuscript, only a comment on novel candidate regulators through rigorous bioinformatic analyses.

I believe that there are findings of interest in this manuscript and with some clarification that a firmer technical foundation can provide, it will be useful to biologists from several fields. If the authors choose not to incoporate these changes, acknowledging these caveats in the writing very plainly and conspicuously will suffice.

Reviewer 3

Advance summary and potential significance to field

While several epigenetic marks clearly regulate early germ cell activity, few have been studied in depth. In this manuscript, the authors sought to explore the role of the H3K4me methyltransferase, Set1, in regulating gametogenesis, a stem cell-supported process, in the Drosophila testis. Due to technical constraints precluding the use of mutant analysis, the authors knocked down Set1 using the UAS/GAL4 system documenting a series of germline and somatic cell phenotypes at several timepoints using multiple driver lines. Impressively, the authors generated multiple GFP-tagged, RNAi-invulnerable rescue transgenes to advance the argument that Set1's methyltransferase activity specifically is required in early-stage germ cells.
To identify likely Set1 targets, the authors conducted RNA-seq analysis of testes with and without Set1 knockdown. While they did not identify likely direct targets of Set1, they noted that both JAK-

© 2024. Published by The Company of Biologists under the terms of the Creative Commons Attribution License (https://creativecommons.org/licenses/by/4.0/).

STAT and BMP signaling pathways—well-known regulators of germ cell maintenance and proliferation—are affected by Set1 knockdown. Nevertheless, reducing either stat92E or mad dosage in the Set1 knockdown background altered Set1 knockdown phenotypes, suggesting that Set1 impinges on processes controlled by JAK-STAT and BMP signaling.

The role of epigenetic modifications in regulating stem cell activity is likely to be of broad interest to the audience of this journal. However, a central and critical weakness of the manuscript is a lack of clarity regarding the Set1 germline knockdown phenotypes. This encompasses several issues: the use of multiple experimental paradigms with conflicting results, a reliance on qualitative phenotypic analysis, and a lack of narrative surrounding the “progressive” nature of the described phenotype. Significant revisions will be necessary to raise the study to the standard of publication in Development.

Comments for the author

Vidaurre et al.; Development submission Summary:

While several epigenetic marks clearly regulate early germ cell activity, few have been studied in depth. In this manuscript, the authors sought to explore the role of the H3K4me methyltransferase, Set1, in regulating gametogenesis, a stem cell-supported process, in the *Drosophila* testis. Due to technical constraints precluding the use of mutant analysis, the authors knocked down Set1 using the UAS/GAL4 system, documenting a series of germline and somatic cell phenotypes at several timepoints using multiple driver lines. Impressively, the authors generated multiple GFP-tagged, RNAi-invulnerable rescue transgenes to advance the argument that Set1’s methyltransferase activity specifically is required in early-stage germ cells. To identify likely Set1 targets, the authors conducted RNA-seq analysis of testes with and without Set1 knockdown. While they did not identify likely direct targets of Set1, they noted that both JAK-STAT and BMP signaling pathways—well-known regulators of germ cell maintenance and proliferation—are affected by Set1 knockdown. Nevertheless, reducing either *stat92E* or *mad* dosage in the Set1 knockdown background altered Set1 knockdown phenotypes, suggesting that Set1 impinges on processes controlled by JAK-STAT and BMP signaling.

The role of epigenetic modifications in regulating stem cell activity is likely to be of broad interest to the audience of this journal. However, a central and critical weakness of the manuscript is a lack of clarity regarding the Set1 germline knockdown phenotypes. This encompasses several issues: the use of multiple experimental paradigms with conflicting results, a reliance on qualitative phenotypic analysis, and a lack of narrative surrounding the “progressive” nature of the described phenotype. Significant revisions will be necessary to raise the study to the standard of publication in *Development*.

Major concerns

The authors claim that early-stage specific knockdown of Set1 leads to “temporally progressed defects” where first, germ cells are lost, then, remaining germ cells become overpopulated in the early stages of spermatogenesis. While the phenotypes that they document are interesting, improvements are necessary to better understand the role of Set1 in the male germline.

- The conclusion that Set1 is required for GSC maintenance is not well-supported by all the data presented in the manuscript. For example, in Fig. 1E, Set1 knockdown testes have *consistently* lower GSCs than control knockdown; this is not a phenotype that appears to be progressing with time. Instead, this phenotype suggests that the number of GSCs in each genotype was different at the point of eclosion, and this difference was maintained over time. Importantly, this phenotype is different in flies that have had Set1 knockdown suppressed by a GAL80 during development. Indeed, Fig. S2D depictsa trend in GSC number that is consistent with GSC maintenance requirement for Set1.
  However, this experiment takes place over a *much different* time course that the

© 2024. Published by The Company of Biologists under the terms of the Creative Commons Attribution License (https://creativecommons.org/licenses/by/4.0/).

data presented in Fig.1. Elsewhere in the manuscript, single timepoints are used to argue for differences in GSC maintenance between genotypes (e.g. Fig. 5); this is not consistent with the way GSC maintenance usually reported in the literature (over time). Further, given the clear differences in H3K4me at day 0 in this knockdown paradigm (Fig. S1), it will be important to demonstrate that these differences arose *after* eclosion.

- Generally speaking, phenotypic differences between the GAL80 and the non-GAL80 experimental paradigms strongly suggest that Set1 could have different roles at different developmental times. The manuscript in its current form barely acknowledges the differences in these phenotypes, much less explain them. In addition to the GSC loss phenotype (discussed above), the hub size phenotype appears different in these different schemes; specifically, the dramatic phenotype appears to have a developmental component given the dampened effect when analyzing flies reared with GAL80ts. While the authors suggest that some of these differences may arise from temperature-specific effects, an interesting possibility, it will be important to clearly tease out adult-specific roles to support their conclusions.
- It would be helpful to readers to understand if “early germ cell overpopulation” arises from compromised differentiation or proliferation (e.g. by directly assaying for proliferation or markers of differentiation).

- The authors state that “changes in cyst cell number appear to coincide with the changes in the germline phenotypes over the duration of the time course in the *set1* KD testes, indicating Set1 acts in the germline to regulate somatic gonadal cells in a non-cell- autonomous manner”. Are they suggesting that the change in somatic support is a likely contributor to the germline “overpopulation”? If so, it would be helpful to see if there is a relationship there (i.e. if germ cell number and cyst cell number are increased to the same extent in the same testis).

The genetic interaction experiments presented in Figure 5 could be improved by including mutant alleles from other pathway constituents. For example, it is not surprising that reducing *stat92E* dosage in a Set1 knockdown background leads to less Stat92E protein; are other manipulations possible? Further, the conclusion that “Set1 regulates key JAK-STAT and BMP signaling components” should be softened. Reducing *mad* dosage in the background of Set germline knockdown alleviates the phenotypes associated with Set1 knockdown. However, given that BMP signaling is a critical regulator of GSC maintenance, it is possible that Set1 is not acting through the BMP signaling pathway, but that these effects are additive. The authors should be careful to clarify this point.

Minor concerns

Page 10, “As controls, three additional transgenes were generated and expressed at the same genetic background”. Do the authors mean locus?

Page 13 “…we examined two key downstream genes of JAK-STAT and BMP pathways, *mad* and *stat92E*, respectively.” The order of *mad* and *stat92E* has been transposed.

Page 14, “…in the *set1* KD testes, pMad can be destected in GSCs and even ectopically in the spermatogonial cells”. A good control here would be control RNAi with pMad stain! Additionally, since the authors see different staining patterns here, it would be helpful if those could be mapped onto the observed phenotypes.

Figure 4 legend: TPM is not defined

First revision

<u>Author response to reviewers' comments</u>

Point-to-point Responses:

© 2024. Published by The Company of Biologists under the terms of the Creative Commons Attribution License (https://creativecommons.org/licenses/by/4.0/).

Reviewer 1:

Proper regulation of stem cell lineages is important for tissue function. Stem cell maintenance and differentiation can be regulated by external as well as internal factors, such as epigenetic regulators. In this manuscript, Vidaurre et al propose that the histone methyltransferase Set1 regulates germline stem cell (GSC) maintenance and progeny differentiation in the adult Drosophila testis. The authors provide evidence that knockdown of Set1 specifically in the germline decreases GSC numbers and that early differentiating cysts overpopulate and display tumor phenotypes. The authors further propose that loss of set1 enhances JAK/STAT and BMP signaling. The findings from this study will be of interest to researchers in the epigenetic, stem cell, and cancer-related fields.

Reviewer 1 Comments for the Author:
I found this manuscript and the findings from the authors extremely interesting; however I do have suggestions and comments regarding statistical analyses, quantification, and proper controls used for the experiments performed in the current version of the manuscript.

We thank this reviewer for the overall very positive comments.
Major points:
1. The statistical analysis using an unpaired t-test does not seem appropriate. Because the different genotypes could have different starting points (e.g., different GSC numbers to begin with), it is unfair to do a direct comparison between the control and set1 RNAi. For the time course experiments, it would be better to perform an ANOVA with interaction. Furthermore, the one timepoint only analyses have the same problem (we don't know where the GSC/cyst cell numbers/etc started out at day 0, so statements suggesting that GSC numbers are higher or lower aren't fully accurate.

We appreciate this reviewer's suggestions. We have re-performed statistical analysis as this reviewer recommended (see revised Figure 1 and legends).

We have now incorporated data from an earlier developmental stage at the third instar larval stage, when stem cells and their niche have already been established (Le Bras and Van Doren, 2006). Our findings show no detectable germ cell loss or early-stage germline over-proliferation phenotypes in *set1* knockdown testes at this stage (see revised Figure 1C and 1D). Other phenotypes, such as germline stem cell number and hub size, show either minimal differences or no significant differences between the control and *set1* knockdown testes, respectively (see revised Figure 1E and 1F). Since the difference in germline stem cell number is already detectable at the third instar larval stage, we conclude that *set1* is required for germline stem cell maintenance in adulthood based on temperature-controlled *set1* RNAi results. We have also moved these data from the Supplemental Information to the Main Figure (Figure 2), where the knockdown occurs specifically in adulthood. These data demonstrate that Set1 is intrinsically required for germline stem cell maintenance in adult testes. We have revised the text accordingly to acknowledge the limitation of each experimental design.

2. Please define the parameters for overpopulation (never defined in the text). How is this determined? Is it based on the number of 2-, 4-, 8-cell cysts per GSC? The authors would need to determine this to say whether or not there are more early stage cysts in the set1 RNAi conditions compared to control.

We used a membrane marker (i.e., anti-Armadillo) for cyst cells which encapsulate germ cells, as shown previously (Feng et al., 2017). Any cyst with more than 16 germ cells are considered overpopulated. We have revised the corresponding text by including this definition.

3. How can the authors determine if the phenotypes are due to over proliferation (e.g., red label in graph for Figure 1C) and not de-differentiation of cysts? Labeling with EdU or pHH3 would need to be performed to say it is over proliferation.

We appreciate this reviewer's comment and we have done these suggested experiments using antibodies against H3S10ph, a mitosis-enriched cellular marker, to show those excessive early-

© 2024. Published by The Company of Biologists under the terms of the Creative Commons Attribution License (https://creativecommons.org/licenses/by/4.0/).

stage cells are indeed over-proliferative. We now include the representative images in a new supplemental figure (Figure S2).

4. The authors suggest that GSC maintenance is affected with set1 is knocked down in the germline during development (Figure 1E). However, if you look at the slopes, the maintenance of the GSCs is not different compared to the control – the different genotypes simply start out at different points. The more convincing data regarding maintenance is found in the supplemental with the adult-specific manipulations. I would recommend either moving the adult analysis to the main figures or re-interpreting the developmental knockdown conclusions.

We agree with this comment and has moved temperature controlled *set1* RNAi results from supplement to main figure (Figure 2), where the knockdown occurs specifically in adulthood. These data demonstrate that Set1 is intrinsically required for germline stem cell maintenance in adult testes.

5. The phenotypes observed with set1 RNAi seem to be more exaggerated when manipulation occurs during development. What do the larval and pupal testis look like compared to control?

We have now incorporated data from earlier developmental stages at the third instar larval stage. Our findings show no detectable germ cell loss or early-stage germline over-proliferation phenotypes in *set1* knockdown testes at this stage (see revised Figure 1C and 1D). Other phenotypes, such as germline stem cell number and hub size, show either minimal differences or no significant differences between the control and *set1* knockdown testes, respectively (see revised Figure 1E and 1F).

6. How do the authors distinguish early cysts from late cysts? The methods say that the early cyst number was quantified by counting every Tj positive cell. Wouldn't that include late cysts?

We thank this reviewer's comments and indeed we have performed immunostaining using antibodies against the Zinc-finger homeodomain protein 1 (Zfh-1), a marker for CySCs and early-stage cyst cells (Eun et al., 2014; Issigonis et al., 2009; Leatherman and Dinardo, 2008) and now include these data in a new supplemental figure (Figure S3).

7. The authors mention in the text (page 10) that differences observed in the phenotypes could be due to differences in knockdown strength. It is unclear why additional RNAi lines were not used (two TRiP lines available in the BDSC, and two different lines available in the VDRC).

We thank this reviewer's comments and we did try the other available TRiP line but unfortunately this line fails to decrease the H3K4me3 levels when expressed in the germ cells, suggesting that it does not effectively knock down *set1*. Therefore, we went ahead and designed a series of rescuing experiments to show that the phenotypes are caused by loss-of-function of the *set1* gene and depends on its methyl-transferase activity (Fig. 3 and Fig. S6).

8. How do the authors quantify/determine "normal morphology" (Figure 5C, page 14)? Some of the nos>set1 RNAi images look similarly "normal."

We used the same criterion to determine the severity and category of the phenotypes, as defined in Figure 1D. We now added these clarifications to the revised Figure 5E legend.

9. For the genetic interaction analysis using the stat92E[06346] and mad[12] alleles, a better control would be to have these alleles in the background of the nos-Gal4 driver (Figure 5).

We thank this reviewer's suggestions and indeed we used the following genetic backgrounds as the control, which were abbreviated in the figure labeling: *nos-Gal4/mad*^{12} (on the 2nd chromosome, abbreviated as *mad*^{12}/+) and *UAS-shSet1/stat92E*^{06346} (on the 3rd chromosome, abbreviated as *stat92E*^{06346}/+). We now added these detailed genotypes to Figure 5A and 5B legend.

10. Fluorescence intensities in Figures 5A and 5B should be quantified for pMad and Stat92E levels.

© 2024. Published by The Company of Biologists under the terms of the Creative Commons Attribution License (https://creativecommons.org/licenses/by/4.0/).

We have added more quantifications of the pMad and Stat92E immunostaining results in the revised figure (see revised Fig. 5C and 5D).

11. The argument that set1 RNAi influences negative regulators of JAK/STAT and BMP signaling would be strengthened if rescue experiments were performed. There are UAS constructs available in the BDSC for Ptp61F and Ube3.

We thank this reviewer for their suggestions. We have performed more genetic interaction experiments for the connections between *set1* and the JAK-STAT or BMP signaling pathways. We have tested *thickveins* (*tkv*), which encodes the upstream receptor of the BMP pathway and displays increased expression in the *set1* KD testes compared to the *Ctrl* KD testes (see revised Fig. S7D). We now include these new results in the revised Figure 5 (see revised Fig.5E-F). These results based on *mad* and *tkv* mutations suggest that Set1 normally represses the BMP signaling pathway. Without this repression, ectopic activity can occur, leading to abnormal germline morphology and composition.
We have also tested lines for *ptp61F*, which encodes a negative regulator of the JAK-STAT pathway, as well as a *cul2* mutant line, which encodes a negative regulator of the BMP pathway. However, the gene expression changes of these two genes in *set1* knockdown testes compared to the control are minimal (~10%) without significant differences (*P*= 0.51 for *ptp61F*; *P*= 0.43 for *cul2*, see revised Fig. S9G). Because of these and potential involvement of either other factors of the JAK-STAT or BMP signaling pathways or components in other signaling pathways (see revised Fig. S7D), these genetic interaction experiments are not conclusive and we did not include them in the revision. We revised the related Results and Discussion to make them more open-ended.

Minor points:
1. Why do the authors only perform two biological replicates instead of at least three?

As shown in Figure S7, we did three independent biological replicates for the RNA-seq experiments for each genotype at each time point. With 3 biological replicates, 2 genotypes and 4 time points, we analyzed all 24 samples in Figure 4 and Figure S7. We have added these details to the corresponding figure legends.

2. The Vasa staining for the 21 and 28 day set1 RNAi images is missing.

This comment should refer to the previous Figure S2 (current Figure 2), where we did anti-Vasa immunostaining for the Day 21 and 28 *set1* RNAi samples. However, there is no Vasa-positive cells and all remaining cells are cyst cells. We apologize for the confusion and have added these details to the figure legend.

<u>Reviewer 2:</u>

In this manuscript by Velinda Vidaurre et al., the authors make an interesting observation regarding different functions of the same gene product, Set1, in different steps of the differentiation of Drosophila germline stem cells. They show that knockdown of Set1 induced in adult germ cells leads first, to the loss of more differentiated germ cells, followed by over-proliferation of the early germ cells themselves. They implicate the Jak-stat and BMP signalling pathway data based on RNA seq data.

We thank this reviewer for the positive comments and constructive suggestions.

Reviewer 2 Comments for the Author:
While the manuscript is convincing at the face of it, I recommend the following additions and amendments for rigour and alleviating doubt:
1. Use of a single RNAI line: In the study, the loss of Set1 is achieved by the use of a single RNAi line.
Formalism requires at least three indelpendent lines with at least one rescue, both of the phenotype and the posited disrupted mechanism. Experiments shown in figure 3 with the catalytically inactive Set1, do suggest that phenotype may be due to Set1b loss of function

© 2024. Published by The Company of Biologists under the terms of the Creative Commons Attribution License (https://creativecommons.org/licenses/by/4.0/).

alone. However, because subsequent analysis is based on bulk RNA-seq of the knockdown tissue, where off target effects will undoubtedly manifest, I suggest the phenotype be reproduced with at least one more RNAi Line and the RNA seq data show agreement with some identified targets. This experiment may be essential in view of transcriptional compensation that has been previously reported.

We thank this reviewer's comments and we did try the other available TRiP line but unfortunately this line fails to decrease the H3K4me3 levels when expressed in the germ cells, suggesting that it does not effectively knock down *set1*. Therefore, we went ahead and designed a series of rescuing experiments to show that the phenotypes are caused by loss-of-function of the *set1* gene and depends on its methyl-transferase activity (Fig. 3 and Fig. S6).

2. JNK and BMP as effectors of Set1 KD phenotype: As the authors note JNK and BMP are known pathways regulating stem cell maintenance. The RNAi KD show great statistically significant differences in their levels upon downregulation. However, the fold changes are between 2 and 4 times. Their assertion is supported by the modification of the set1 KD phenotype being modified by stat92E alleles. The modification, though, is cell clearance, which may be cell death. Many perturbations can enhance the clearance of cells, so the assay for epistasis analysis may not be well-founded.
In this regard, I have two suggestions: i) RNAseq of KD tissue from a second independent RNAi line to check if the same targets filter through (same experiment as the previous point). ii) At least an analysis of other candidate regulatory pathways so that the field may go beyond the known pathways that have been dwelled on for decades. No experimental verification is needed for this manuscript, only a comment on novel candidate regulators through rigorous bioinformatic analyses.

We thank this reviewer for the insightful suggestions. Based on the RNA-seq data, we have identified components of several other signaling pathways, such as EGF, Notch, Hedgehog, Wnt, and Hippo pathways, that have differential gene expression in the *set1* knockdown samples compared to the control samples. We now add a revised Table (see revised Fig. S7D) and more discussion related to these findings. Further experiments will be needed to address whether they are potentially novel regulators of the *Drosophila* early male germline but these would be beyond the current manuscript, as this reviewer commented.

I believe that there are findings of interest in this manuscript and with some clarification that a firmer technical foundation can provide, it will be useful to biologists from several fields. If the authors choose not to incoporate these changes, acknowledging these caveats in the writing very plainly and conspicuously will suffice.

We also revised the manuscript to acknowledge the limitation and make it more inclusive.

<u>Reviewer 3:</u>

While several epigenetic marks clearly regulate early germ cell activity, few have been studied in depth. In this manuscript, the authors sought to explore the role of the H3K4me methyltransferase, Set1, in regulating gametogenesis, a stem cell-supported process, in the Drosophila testis. Due to technical constraints precluding the use of mutant analysis, the authors knocked down Set1 using the UAS/GAL4 system, documenting a series of germline and somatic cell phenotypes at several timepoints using multiple driver lines. <u>Impressively</u>, the authors generated multiple GFP-tagged, RNAi-invulnerable rescue transgenes to advance the argument that Set1's methyltransferase activity specifically is required in early-stage germ cells. To identify likely Set1 targets, the authors conducted RNA-seq analysis of testes with and without Set1 knockdown. While they did not identify likely direct targets of Set1, they noted that both JAK-STAT and BMP signaling pathways—well-known regulators of germ cell maintenance and proliferation—are affected by Set1 knockdown. Nevertheless, reducing either stat92E or mad dosage in the Set1 knockdown background altered Set1 knockdown phenotypes, suggesting that Set1 impinges on processes controlled by JAK-STAT and BMP signaling.
The role of epigenetic modifications in regulating stem cell activity is likely to be of broad interest to the audience of this journal. However, a central and critical weakness of the manuscript is a lack of clarity regarding the Set1 germline knockdown phenotypes. This

© 2024. Published by The Company of Biologists under the terms of the Creative Commons Attribution License (https://creativecommons.org/licenses/by/4.0/).

encompasses several issues: the use of multiple experimental paradigms with conflicting results, a reliance on qualitative phenotypic analysis, and a lack of narrative surrounding the “progressive” nature of the described phenotype. Significant revisions will be necessary to raise the study to the standard of publication in Development.

We thank this reviewer for the positive comments and constructive suggestions.
Major concerns
The authors claim that early-stage specific knockdown of Set1 leads to “temporally progressed defects” where first, germ cells are lost, then, remaining germ cells become overpopulated in the early stages of spermatogenesis. While the phenotypes that they document are interesting, improvements are necessary to better understand the role of Set1 in the male germline.
The conclusion that Set1 is required for GSC maintenance is not well-supported by all the data presented in the manuscript. For example, in Fig. 1E, Set1 knockdown testes have consistently lower GSCs than control knockdown; this is not a phenotype that appears to be progressing with time. Instead, this phenotype suggests that the number of GSCs in each genotype was different at the point of eclosion, and this difference was maintained over time. Importantly, this phenotype is different in flies that have had Set1 knockdown suppressed by a GAL80 during development. Indeed, Fig. S2D depictsa trend in GSC number that is consistent with GSC maintenance requirement for Set1. However, this experiment takes place over a much different time course that the data presented in Fig.1. Elsewhere in the manuscript, single timepoints are used to argue for differences in GSC maintenance between genotypes (e.g. Fig. 5); this is not consistent with the way GSC maintenance usually reported in the literature (over time). Further, given the clear differences in H3K4me at day 0 in this knockdown paradigm (Fig. S1), it will be important to demonstrate that these differences arose after eclosion.
Generally speaking, phenotypic differences between the GAL80 and the non-GAL80 experimental paradigms strongly suggest that Set1 could have different roles at different developmental times. The manuscript in its current form barely acknowledges the differences in these phenotypes, much less explain them. In addition to the GSC loss phenotype (discussed above), the hub size phenotype appears different in these different schemes; specifically, the dramatic phenotype appears to have a developmental component given the dampened effect when analyzing flies reared with GAL80ts. While the authors suggest that some of these differences may arise from temperature-specific effects, an interesting possibility, it will be important to clearly tease out adult-specific roles to support their conclusions.

We appreciate this reviewer’s suggestions. We have now incorporated data from an earlier developmental stage at the third instar larval stage, when stem cells and their niche have already been established (Le Bras and Van Doren, 2006). Our findings show no detectable germ cell loss or early-stage germline over-proliferation phenotypes in *set1* knockdown testes at this stage (see revised Figure 1C and 1D). Other phenotypes, such as germline stem cell number and hub size, show either minimal differences or no significant differences between the control and *set1* knockdown testes, respectively (see revised Figure 1E and 1F). Since the difference in germline stem cell number is already detectable at the third instar larval stage, we conclude that *set1* is required for germline stem cell maintenance in adulthood based on temperature-controlled *set1* RNAi results. We have also moved these data from the Supplemental Information to the Main Figure (Figure 2), where the knockdown occurs specifically in adulthood. These data demonstrate that Set1 is intrinsically required for germline stem cell maintenance in adult testes. We have revised the text accordingly to acknowledge the limitation of each experimental design.

It would be helpful to readers to understand if “early germ cell overpopulation” arises from compromised differentiation or proliferation (e.g. by directly assaying for proliferation or markers of differentiation).

We appreciate this reviewer’s comment and we have done these suggested experiments using antibodies against H3S10ph, a mitosis-enriched cellular marker, to show those excessive early-stage cells are indeed over-proliferative. We now include the representative images in a new supplemental figure (Figure S2).

The authors state that “changes in cyst cell number appear to coincide with the changes in the germline phenotypes over the duration of the time course in the set1 KD testes, indicating Set1 acts in the germline to regulate somatic gonadal cells in a non-cell-autonomous manner”. Are

© 2024. Published by The Company of Biologists under the terms of the Creative Commons Attribution License (https://creativecommons.org/licenses/by/4.0/).

they suggesting that the change in somatic support is a likely contributor to the germline "overpopulation"? If so, it would be helpful to see if there is a relationship there (i.e. if germ cell number and cyst cell number are increased to the same extent in the same testis).

We hypothesize that the knockdown of *set1* in germ cells lead to those phenotypic changes in cyst stem cell number, in a non-cell-autonomous manner. We now clarify it in the Abstract and Result parts.

The genetic interaction experiments presented in Figure 5 could be improved by including mutant alleles from other pathway constituents. For example, it is not surprising that reducing stat92E dosage in a Set1 knockdown background leads to less Stat92E protein; are other manipulations possible? Further, the conclusion that "Set1 regulates key JAK-STAT and BMP signaling components" should be softened. Reducing mad dosage in the background of Set germline knockdown alleviates the phenotypes associated with Set1 knockdown. However, given that BMP signaling is a critical regulator of GSC maintenance, it is possible that Set1 is not acting through the BMP signaling pathway, but that these effects are additive. The authors should be careful to clarify this point.

We thank this reviewer for their suggestions. We have performed more genetic interaction experiments for the connections between *set1* and the JAK-STAT or BMP signaling pathways. We have tested *thickveins* (*tkv*), which encodes the upstream receptor of the BMP pathway and displays increased expression in the *set1* KD testes compared to the *Ctrl* KD testes (see revised Fig. S7D). In all genetic interaction assays, we tested that heterozygous *stat92E*, *mad*, and *tkv* do not lead to any detectable phenotypes (see revised Fig. 5E-F). We now include these new results in the revised Figure 5 (see revised Fig.5E-F). These results based on *mad* and *tkv* mutations suggest that Set1 normally represses the BMP signaling pathway. Without this repression, ectopic activity can occur, leading to abnormal germline morphology and composition. We also revised the related Results and Discussion to make them more open-ended.

Additionally, based on the RNA-seq data, we have identified components of several other signaling pathways, such as EGF, Notch, Hedgehog, Wnt, and Hippo pathways, that have differential gene expression in the *set1* knockdown samples compared to the control samples. We now add a revised Table (see revised Fig. S7D) and more discussion related to these findings. Further experiments will be needed to address whether they are potentially novel regulators of the *Drosophila* early male germline but these would be beyond the current manuscript.

Minor concerns
Page 10, "As controls, three additional transgenes were generated and expressed at the same genetic background". Do the authors mean locus?

By genetic background we meant that each transgene line was crossed individually to the same *set1* RNAi background to perform rescuing experiments. We apologize for any confusion and now clarify this point in the revision.

Page 13 "…we examined two key downstream genes of JAK-STAT and BMP pathways, mad and stat92E, respectively." The order of mad and stat92E has been transposed.

We thank this reviewer for noticing this and we have corrected it now.

Page 14, "…in the set1 KD testes, pMad can be destected in GSCs and even ectopically in the spermatogonial cells". A good control here would be control RNAi with pMad stain! Additionally, since the authors see different staining patterns here, it would be helpful if those could be mapped onto the observed phenotypes.

We thank this reviewer's suggestions and indeed we used the following genetic backgrounds as the control, which were abbreviated in the figure labeling: *nos-Gal4/mad*^{12} (on the 2nd chromosome, abbreviated as *mad*^{12}/+) and *UAS-shSet1/stat92E*^{06346} (on the 3rd chromosome, abbreviated as *stat92E*^{06346}/+). We now added these detailed genotypes to Figure 5A and 5B legend.

© 2024. Published by The Company of Biologists under the terms of the Creative Commons Attribution License (https://creativecommons.org/licenses/by/4.0/).

We also thank this reviewer for additional controls. We have performed more control immunostaining experiments using antibodies against Stat92E and pMad, the key downstream factors for the JAK-STAT or BMP signaling pathways, respectively. These new controls include *nos>mCherry RNAi* testes (*nos>Control RNAi* or *Ctrl* KD), which show similar patterns as the other controls in Figure 5. We now include the representative images in a new supplemental figure (Fig. S8).

Finally, we have added more quantifications of the pMad and Stat92E immunostaining results in the revised figure (see revised Fig. 5C and 5D).

Figure 4 legend: TPM is not defined

It is now defined: Transcript Per Million reads (see Methods).

References:

Eun, S.H., Shi, Z., Cui, K., Zhao, K., Chen, X., 2014. A non-cell autonomous role of E(z) to prevent germ cells from turning on a somatic cell marker. Science 343, 1513-1516.
Feng, L., Shi, Z., Chen, X., 2017. Enhancer of polycomb coordinates multiple signaling pathways to promote both cyst and germline stem cell differentiation in the Drosophila adult testis. PLoS Genet 13, e1006571.
Issigonis, M., Tulina, N., de Cuevas, M., Brawley, C., Sandler, L., Matunis, E., 2009. JAK- STAT signal inhibition regulates competition in the Drosophila testis stem cell niche. Science 326, 153-156.
Le Bras, S., Van Doren, M., 2006. Development of the male germline stem cell niche in Drosophila. Dev Biol 294, 92-103.
Leatherman, J.L., Dinardo, S., 2008. Zfh-1 controls somatic stem cell self-renewal in the Drosophila testis and nonautonomously influences germline stem cell self-renewal. Cell Stem Cell 3, 44-54.

Second decision letter

MS ID#: DEVELOP/2024/202729

MS TITLE: The *Drosophila* histone methyl-transferase SET1 coordinates multiple signaling pathways in regulating male germline stem cell maintenance and differentiation

AUTHORS: Velinda Vidaurre, Annabelle Song, Taibo Li, Wai Lim Ku, Keji Zhao, Jiang Qian, and Xin Chen

I am happy to tell you that your manuscript has been accepted for publication in Development, pending our standard publication integrity checks. If you like to modify the text according to a concern given by the reviewer 1 , this could be accepted though is not compulsory.

Reviewer 1

Advance summary and potential significance to field

Proper regulation of stem cell lineages is important for tissue function. Stem cell maintenance and differentiation can be regulated by external as well as internal factors, such as epigenetic regulators. In this manuscript, Vidaurre et al propose that the histone methyltransferase Set1 regulates germline stem cell (GSC) maintenance and progeny differentiation in the adult Drosophila testis. The authors provide evidence that knockdown of Set1 specifically in the germline decreases GSC numbers and that early differentiating cysts overpopulate and display tumor phenotypes. The authors further propose that loss of set1 enhances JAK/STAT and BMP signaling. The findings from this study will be of interest to researchers in the epigenetic, stem cell, and cancer-related fields.

Comments for the author

© 2024. Published by The Company of Biologists under the terms of the Creative Commons Attribution License (https://creativecommons.org/licenses/by/4.0/).

The authors have sufficiently addressed all of my concerns from the previous submission. I do not have any additional comments or suggestions that would strengthen the study.

Reviewer 2

Advance summary and potential significance to field

In this manuscript by Velinda Vidaurre et al., the authors make an interesting observation regarding different functions of the same gene product, Set1, in different steps of the differentiation of Drosophila germline stem cells.
They show that knockdown of Set1 induced in adult germ cells leads first, to the loss of more differentiated germ cells, followed by over-proliferation of the early germ cells themselves. They implicate the Jak-stat and BMP signalling pathway data based on RNA seq data.

Comments for the author

All my concerns have been satisfactorily addressed.

Reviewer 3

Advance summary and potential significance to field

The authors have thoughtfully addressed many reviewer concerns, including all of my major concerns, with this revision.

Comments for the author

I am particularly pleased with the authors' decision to move temperature sensitive manipulations out of the supplement, which significantly clarifies the GSC maintenance phenotype. However, I am confused by this statement:

"Phenotypes such as germline stem cell number and hub size show much less or no significant difference between the set1 KD and Ctrl KD testes, respectively (Fig. 1E-F)"

According to the data presented in Figure 1, germline stem cell number and the number of TJ-positive cells is different in control and experimental groups when some knockdown is permitted during development. Since a role in development for Set1 does not preclude a role for Set1 in adult GSC maintenance, I don’t think the authors should be reluctant to plainly state this. Of course, the authors go on to show a much more robust phenotype with longer knockdown. This is a minor concern, but considering the number of pathways that interact to support GSC maintenance, these small differences may be physiologically relevant. Further, other groups may find the developmental differences to be interesting.

© 2024. Published by The Company of Biologists under the terms of the Creative Commons Attribution License (https://creativecommons.org/licenses/by/4.0/).
